# Chemical exposure-response relationship between air pollutants and reactive oxygen species in the human respiratory tract

**DOI:** 10.1038/srep32916

**Published:** 2016-09-08

**Authors:** Pascale S. J. Lakey, Thomas Berkemeier, Haijie Tong, Andrea M. Arangio, Kurt Lucas, Ulrich Pöschl, Manabu Shiraiwa

**Affiliations:** 1 Max Planck Institute for Chemistry, Multiphase Chemistry Department, Hahn-Meitner-Weg 1, 55128 Mainz, Germany; 2 Department of Chemistry, University of California Irvine, Irvine, CA 92697, USA

## Abstract

Air pollution can cause oxidative stress and adverse health effects such as asthma and other respiratory diseases, but the underlying chemical processes are not well characterized. Here we present chemical exposure-response relations between ambient concentrations of air pollutants and the production rates and concentrations of reactive oxygen species (ROS) in the epithelial lining fluid (ELF) of the human respiratory tract. In highly polluted environments, fine particulate matter (PM2.5) containing redox-active transition metals, quinones, and secondary organic aerosols can increase ROS concentrations in the ELF to levels characteristic for respiratory diseases. Ambient ozone readily saturates the ELF and can enhance oxidative stress by depleting antioxidants and surfactants. Chemical exposure-response relations provide a quantitative basis for assessing the relative importance of specific air pollutants in different regions of the world, showing that aerosol-induced epithelial ROS levels in polluted megacity air can be several orders of magnitude higher than in pristine rainforest air.

Anthropogenic air pollution leads to a massive increase of atmospheric aerosol and oxidant concentrations on local, regional, and global scales, posing a major threat to public health^1^,^2^. The concentrations of fine particulate matter in polluted urban air are several orders of magnitude higher than in pristine air (~10–1000 μg m^−3^ vs. ~1–10 μg m^−3^)^2^, and high pollutant levels can cause serious respiratory and cardiovascular diseases, leading to elevated mortality^3^,^4^,^5^,^6^. For example, mortality rates in the 90 largest U.S. cities were found to rise on average by 0.5% with each 10 μg m^−3^ increase in fine particulate matter^7^, and globally the annual number of premature deaths due to air pollution are estimated to exceed 3 million with an increasing trend^8^. Fine air particulate matter contains redox-active components like transition metals and quinones originating from gasoline and diesel motor exhaust, cigarette smoke, and other sources including secondary organic aerosol (SOA) formation in the atmosphere^9^,^10^,^11^. Upon inhalation and deposition in the human respiratory tract, such air pollutants can induce and sustain chemical reactions that produce reactive oxygen species (ROS; OH, O_2_^−^, HO_2_, O_3_, and H_2_O_2_) in the epithelial lining fluid (ELF) covering the airways^12^. The ELF contains a range of antioxidants and surfactants^13^ (Supplementary information, Supplementary Tables 1 and 3), and it extends from the nasal cavity to the pulmonary alveoli with a film thickness that decreases from several micrometers in the upper airways to dozens of nanometers in the lungs^14^.

As illustrated in Fig. 1, the redox-active pollutants and ROS undergo a multitude of radical and redox reaction cycles in the ELF and the initial step is the transfer of electrons from antioxidants to transition metal ions or quinones forming reduced metal ions or semiquinones, respectively^2^,^11^,^15^. The redox-active transition metal ions and quinones are regenerated by reaction with O_2_ forming O_2_^−^ radicals that are further converted into hydrogen peroxide, which is central to radical reaction cycles and oxidative stress in the respiratory tract^16^. OH radicals, the most reactive form of ROS, can be produced via Fenton-like reactions of H_2_O_2_ with iron or copper ions^17^ and can also be released upon interaction of SOA with water^18^,^19^. Numerous studies have shown that excess ROS can cause oxidative stress injuring cells and tissues in the respiratory tract^2^,^16^. Thus, characterizing the formation of ROS in the ELF is crucial for understanding how air pollution leads to adverse health effects like asthma, allergies and other respiratory diseases.

The production rate and concentration of ROS induced by air pollutants in the ELF, however, have hardly been quantified so far^2^,^12^,^14^. For this purpose we have developed a kinetic multi-layer model of surface and bulk chemistry in the epithelial lining fluid (KM-SUB-ELF), which explicitly treats mass transport and chemical reactions involving air pollutants, ROS, antioxidants and surfactants as detailed in the supplement (Supplementary Fig. 1. & Supplementary Table 1)^20^. It can reproduce experimental data available on the formation and concentrations of H_2_O_2_^15^ and OH^17^ in surrogate ELF containing quinones, iron and copper ions (Supplementary Fig. 2). Characteristic concentration levels of air pollutants in the ELF can be derived from ambient concentrations, breathing rates, and deposition rates in the respiratory tract as detailed in the supplement^15^. With this approach and the KM-SUB-ELF model, we obtained chemical exposure-response relations between the production rates and concentrations of ROS in the ELF and the ambient concentrations and composition of fine particles with diameters <2.5 μm (PM2.5) characteristic for a wide range of geographic locations with different levels of air pollution (Supplementary Tables 4–6).

Figure 2A shows ROS production rates calculated for each location and each redox-active component plotted against the ambient concentration of PM2.5. The data points fall into corridors outlining the relative importance of different chemical components for ROS production in the ELF. The highest ROS production rates are caused by copper and iron ions followed by quinones and SOA. As illustrated in Fig. 2B, the far most abundant form of ROS in the ELF are H_2_O_2_ molecules, which are three to four orders of magnitude more abundant than the O_2_^−^ radicals initially produced from molecular oxygen, six to seven times more abundant than the HO_2_ radicals formed by proton transfer to O_2_^−^, and approximately ten orders of magnitude more abundant than the OH radicals formed by Fenton-like reactions of H_2_O_2_ (Fig. 1). The wide range of concentration levels reflects the vastly different chemical reactivities and lifetimes of the different ROS species. Due to its relatively low reactivity and decomposition rates, H_2_O_2_ is a reservoir species with a chemical lifetime of several hours, whereas the lifetime of OH radicals is less than a microsecond due to their rapid reactions with antioxidants.

The total concentration of ROS generated by redox-active components of PM2.5 deposited in the ELF ranges from ~10 nmol L^−1^ under clean conditions up to almost ~250 nmol L^−1^ under highly polluted conditions as shown in Fig. 2C. The data points are a subset of the data points in Fig. 2A,B, representing the locations for which we could obtain measurement data or well-founded estimates for the characteristic concentration of each of the redox-active components (Supplementary Tables 4–7). The upper and lower bounds of the grey-shaded envelope around the data points are constrained by the approximate upper and lower limit mass fractions and water-soluble fractions of redox-active components typically observed in ambient PM2.5 (see Supplementary section 2).

The green-striped horizontal bar in Fig. 2C indicates ROS concentration levels characteristic for the ELF or bronchoalveolar lavage of healthy humans, respectively, which are around ~100 nmol L^−1^^21^,^22^. Compared to this reference level, the ROS concentrations generated by redox-active particulate matter inhaled from pristine marine or rainforest air (PM2.5 ≪ 10 μg m^−3^) are much lower and appear negligible with regard to airway oxidative stress (ROS < 50 nmol L^−1^). In moderately polluted air (PM2.5 ≈ 10–50 μg m^−3^), the particle-generated ROS concentrations can be of similar magnitude or higher than the physiological background level (ROS ≈ 50–200 nmol L^−1^) and may thus significantly contribute to oxidative stress depending on aerosol concentration and chemical composition. In heavily polluted air (PM2.5 >≈ 50 μg m^−3^), the particle-generated ROS concentrations are as high as the ROS concentrations observed in the bronchoalveolar lavage of patients with acute inflammatory diseases in respiratory tract (ROS ≈ 100–250 nmol L^−1^^21^,^22^). The pathologically high ROS concentrations calculated for the ELF in airways exposed to high ambient aerosol concentrations are consistent with epidemiology-based air quality standards and regulations of the World Health Organization (WHO) and various national environmental protection agencies aiming at PM2.5 concentrations less than ~20–40 μg m^−3^ averaged over one day and less than ~10–20 μg m^−3^ averaged over one year^3^,^4^,^5^.

For selected geographic locations covering a wide range of PM2.5 concentration levels, Fig. 2D shows how the particle-generated ROS concentrations in the ELF would change in response to reducing the concentration of individual or multiple redox-active components by 50%. At all locations strongly influenced by anthropogenic air pollution, the reduction of copper would have the largest effect and reduce the total ROS concentration by ~20%, while the reduction of SOA would decrease total ROS by only ~5% (Supplementary Table 7). Our results are consistent with recent experimental studies emphasizing the high potential of copper to cause oxidative stress and adverse health effects^23^,^24^.

The reduction of iron would decrease ROS by ~10% in moderately polluted air (PM2.5 ≈ 10–50 μg m^−3^). For extremely polluted air (PM2.5 > 100 μg m^−3^), on the other hand, we found that particle-generated ROS levels would even increase upon removal of iron. This is because iron is much more efficient in catalyzing decomposition of H_2_O_2_ to OH radicals (Fenton reaction), while copper and iron are both contributing in similar ways to ROS production. Indeed iron dominates the production of OH radicals (Supplementary Figs 4 and 5), and the reduction of iron leads to a much stronger decrease of OH concentrations (up to ~40%) than the reduction of copper (Supplementary Fig. 6). Thus, reducing iron may still be important for decreasing oxidative stress caused by OH radicals.

Sensitivity studies indicate that Cu and Fe are the redox-active aerosol components most important for ROS production upon inhalation of PM2.5 in polluted regions even if the soluble fractions of the metals are assumed to be low (Supplementary Fig. 3). Thus, we suggest that the emission of copper- and iron-containing particles should be considered as a major target of air pollution control with regard to oxidative stress in the human respiratory tract. Automobile brake and tire wear are major sources of Cu and Fe in polluted air^25^, and reducing these emissions might help to reducing oxidative stress and the adverse health effects related to air particulate matter and road traffic especially in densely populated regions. Correlations with inflammatory and oxidative stress parameters have also been reported for other water-soluble metal ions such as Zn and As^26^,^27^. Both the concentrations and the soluble fractions of metal ions in PM2.5 should thus be more widely monitored to gather comprehensive input for epidemiological studies assessing the reasons and mechanisms of aerosol health effects and oxidative stress in the respiratory system. Due to air exchange, the concentrations and composition of PM2.5 in indoor air tends to be similar to outdoor air^28^,^29^. During periods of activity such as cooking, cleaning and smoking, however, indoor concentrations of PM2.5 can be much higher^30^, and metal ions can also be released from indoor sources like vacuum cleaners, blenders, food processors, and laser printers^29^. Thus, the inhalation of metal-containing particles is likely also relevant for the health effects of indoor air pollution.

Our findings demonstrate that the complex radical and redox reaction cycles outlined above (Fig. 1) can lead to non-linear changes of ROS concentrations in the ELF in response to concentration changes or removal of individual redox-active components of PM2.5. Thus, we suggest and intend to further advance and apply numerical and experimental techniques for the determination of chemical exposure-response relations for the design of efficient control strategies against adverse health effects of aerosols from different sources.

The understanding and characterization of non-linear interactions also play an important role in controlling the formation and effects of gaseous atmospheric photooxidants that may contribute to oxidative stress in the respiratory tract. Our model results show that OH radicals inhaled with ambient air are rapidly scavenged by surfactants and antioxidants, whereas the ELF is readily saturated with ozone approaching Henry’s law equilibrium concentrations (Supplementary Fig. 7). Figure 2B shows the relative abundance of O_3_ compared to other ROS species, indicating that the O_3_ concentration is higher than O_2_^−^ concentrations but less than H_2_O_2_ concentrations. Nevertheless, elevated ozone can contribute to oxidative stress by depleting antioxidants and surfactants in the ELF: As shown in Fig. 3, an increase of ozone from typical background concentration levels (~30 ppb) to summer smog conditions (>≈100 ppb) reduces the chemical half-life from days to hours for antioxidants and from hours to minutes for surfactants, which may be comparable or shorter than the physiological replenishment rates^31^. In contrast, the H_2_O_2_-dominated ROS concentrations generated upon inhalation of PM2.5 do not substantially reduce the chemical half-life and concentration of antioxidants and surfactants (Supplementary Fig. 8). Thus, polluted air combining high PM2.5 and high ozone concentrations is expected to cause particularly high oxidative stress with high levels of ROS and low levels of antioxidant and surfactant concentrations in the ELF. Moreover, some of the surfactant ozonation products may act as transduction molecules to trigger the release of endogenous mediators of inflammation^32^, and the ozonolysis of antioxidants may lead to formation of long-lived reactive oxygen intermediates^33^ including the harmful ascorbate ozonide, especially at the low pH levels characteristic for patients with respiratory diseases^34^,^35^,^36^ (Supplementary Figs 9 and 10).

In the course of the Anthropocene^37^, the average mixing ratios of ozone in continental background air have increased by factors of 2–4 from around 10–20 ppb from the beginning of the 19^th^ century to 30–40 ppb in the 21^st^ century^1^,^2^. In urban areas, ozone often reaches ~80–100 ppb during summer and can exceed 200 ppb during smog periods^1^,^38^. As ozone essentially saturates the ELF according to Henry’s law, an increase of ambient ozone translates into an almost proportional increase of ozone concentration in the ELF depleting antioxidants and surfactants in the respiratory tract. Thus, the global increase of both ozone and fine particulate matter in the air of megacities and other densely populated regions around the world constitutes a major threat to public health in the Anthropocene^2^.

The exposure-response relations determined in this study represent a chemical baseline for the primary chemical production of exogenous ROS and oxidative stress upon inhalation and deposition of redox-active air pollutants in the human respiratory tract. On top of this baseline, air pollutants can cause secondary production of endogenous ROS via biological interactions and responses of the human immune system, including the activation of macrophages, mitochondria and ROS-producing enzymes like NADPH-oxidase (“ROS-induced ROS”)^16^,^39^,^40^ or infections and microbial growth induced by biological and nutrient-rich particles^41^. Pollution-generated exogenous ROS may also form damage associated molecular patterns and trigger immune reactions leading to acute or chronic inflammation, e.g., through the toll-like receptor radical cycle^42^. These and other feedback loops may enhance or dampen the oxidative stress caused by the primary production of ROS from redox-active particulate matter, ozone and other atmospheric oxidants like nitrogen oxides. Further experimental investigations and model studies will be required to fully unravel and quantify the adverse health effects of air pollution. The modeling approach and exposure-response relations presented here provide a basis for such investigations and can help to identify key species and processes, such as the cycling of copper ions, to be addressed in further studies and in the advancement of target-specific regional air quality control strategies.

## Methods

KM-SUB-ELF is based on the kinetic multi-layer model for aerosol surface and bulk chemistry (KM-SUB)^20^. The model treats the following processes explicitly: gas-phase diffusion, adsorption and desorption from the surface, bulk diffusion as well as chemical reactions at the surface and in the bulk. The ELF is split into different layers: a sorption layer, a surfactant layer, a near surface bulk layer and a number of bulk layers. The temporal evolution and concentration profile of the various reactants and products can be simulated by solving a set of ordinary differential equations, which describe mass balance of each species by mass transport fluxes and rates of chemical production and loss. The model includes reactions of O_3_ and OH with antioxidants and surfactants, reactions involving quinones, Fenton chemistry of iron ions, Fenton-like chemistry involving copper ions, and HO_x_ chemistry. Please see the supplementary information for detailed chemistry and kinetic parameters as well as determination of ROS concentrations and production rates in the ELF.

## Additional Information

**How to cite this article**: Lakey, P. S. J. *et al.* Chemical exposure-response relationship between air pollutants and reactive oxygen species in the human respiratory tract. *Sci. Rep.* **6**, 32916; doi: 10.1038/srep32916 (2016).

## Supplementary Material

Supplementary Information

## Figures and Tables

**Figure 1:**
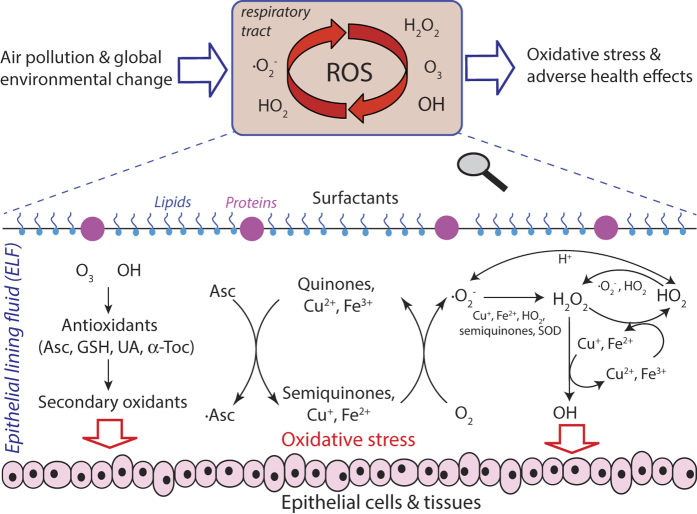
Interaction of air pollutants and reactive oxygen species (ROS) in the epithelial lining fluid (ELF) of the human respiratory tract. ELF can be regarded as an interface between atmospheric and physiological chemistry, through which air pollution and environmental change can induce oxidative stress and adverse health effects. Atmospheric ozone and OH radicals react with surfactants and antioxidants (ascorbate, uric acid, reduced glutathione, α-tocopherol) forming secondary organic oxidants. Redox-active components of fine particulate matter, including quinones, iron and copper ions, can trigger and sustain catalytic reaction cycles generating ROS and oxidative stress.

**Figure 2:**
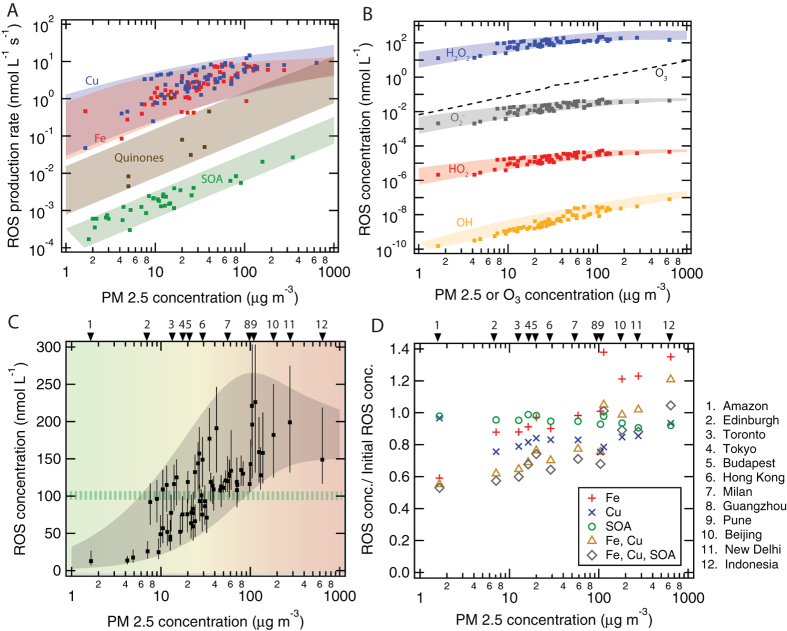
Chemical exposure-response relations for reactive oxygen species (ROS) produced in the human respiratory tract upon inhalation of fine particulate matter (PM2.5). It is shown as a function of PM2.5 concentrations with redox-active components as observed at various geographic locations around the world (Supplementary Tables 4–7). (**A**) ROS production rates induced by copper (Cu), iron (Fe), secondary organic aerosol (SOA), and quinones. (**B**) Characteristic concentration levels of different types of ROS and (**C**) total ROS concentration in the epithelial lining fluid after two hours of inhalation and deposition of ambient PM2.5. In panel (**C**), the green-striped horizontal bar indicates the ROS level characteristic for healthy humans (~100 nmol L^−1^), and the gray envelope represents the range of aerosol-induced ROS concentrations obtained with the approximate upper and lower limit mass fractions of redox-active components typically observed in ambient PM2.5. Total water-soluble fractions of iron and copper can range from ~5–25% and ~20–60%, respectively, in a wide range of different environments, which are represented by the error bars. (**D**) Fractional change of ROS concentrations upon removal of 50% of redox-active components from PM2.5 calculated for selected geographic locations with different PM2.5 concentration levels and composition (Supplementary Table 7).

**Figure 3:**
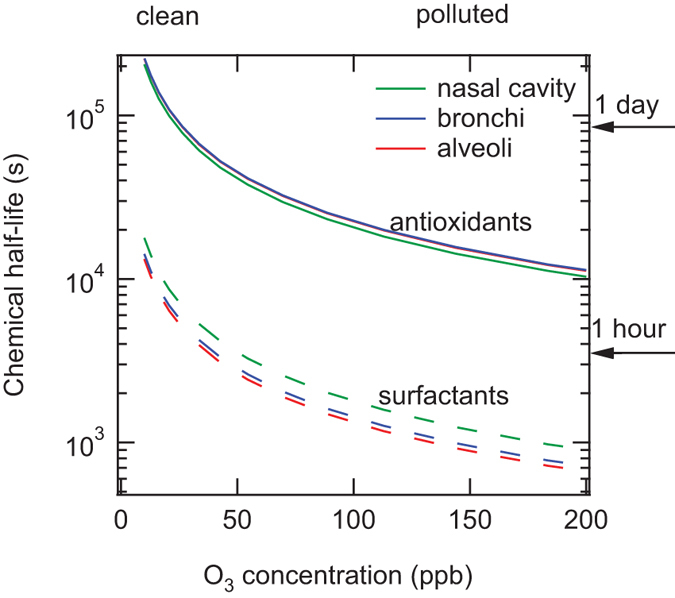
Chemical half-life of antioxidants and surfactants in epithelial lining fluid. They were calculated for the nasal cavity (green), bronchi (blue), and alveoli (red) as a function of ambient ozone concentrations.
