# Supplementary material for: Chemical exposure-response relationship between air pollutants and reactive oxygen species in the human respiratory tract
Source: Sci Rep. 2016 Sep 8;6:32916. doi: 10.1038/srep32916 (PMC5015057; doi:10.1038/srep32916)
Supplement: Supplementary Information [file srep32916-s1.pdf]

Supplementary information for the manuscript "Chemical exposure-response relationship between air pollutants and reactive oxygen species in the human respiratory tract"

Pascale S. J. Lakey[1], Thomas Berkemeier[1], Haijie Tong[1], Andrea M. Arangio[1], Kurt Lucas[1], Ulrich Pöschl[1,*] and Manabu Shiraiwa[1,2*]

[1] Max Planck Institute for Chemistry, Multiphase Chemistry Department, Hahn-Meitner-Weg 1, 55128 Mainz, Germany

[2] Department of Chemistry, University of California Irvine, Irvine, CA 92697, USA

[*] m.shiraiwa@uci.edu; u.poschl@mpic.de

S1. Kinetic model.

KM-SUB-ELF is based on the kinetic multi-layer model for aerosol surface and bulk chemistry (KM-SUB)[1]. The model treats the following processes explicitly: gas-phase diffusion, adsorption and desorption from the surface, bulk diffusion as well as chemical reactions at the surface and in the bulk. The ELF is split into different layers: a sorption layer, a surfactant layer, a near surface bulk layer and a number of bulk layers. In this study 20 bulk layers were used which allowed the concentration gradients within the ELF to be resolved. Laminar flow through the nasal cavity and respiratory tract is expected at resting breathing flow rates[2]. The temporal evolution and concentration profile of the various reactants and products can be simulated by solving a set of ordinary differential equations, which describe mass balance of each species by mass transport fluxes and rates of chemical production and loss. A general schematic of the KM-SUB-ELF model is shown in Supplementary Figure 1.

Supplementary Table 1 summarizes the reactions treated in KM-SUB-ELF. These reactions include O_3 and OH reacting with antioxidants and with a surfactant lipid, 1-palmitoyl-2-oleoyl-*sn*-glycerol (POG) and a surfactant protein (SP-$B_{1\text{-}25}$). POG is representative of the major unsaturated anionic lipids in lung surfactants and contains one double bond that can react with ozone, whereas SP-$B_{1\text{-}25}$ is a protein containing 25 amino acids and is representative of proteins found in lung surfactants. KM-SUB-ELF includes reactions involving three types of quinones, which were found to be the most important in ROS production[3]: phenanthrenequinone (PQN), 1,4-naphthoquinone (1,4-NQN) and 1,2-naphthoquinone (1,2-NQN). KM-SUB-ELF also includes Fenton chemistry of iron ions, Fenton-like chemistry involving copper ions, and HO_x chemistry, leading to both H_2O_2 production and destruction. For the majority of reactions rate coefficients are known and have been published in the literature. For unknown and controversial reaction rate coefficients, the experimental data of formation of H_2O_2[3] and

OH[4] in the presence of quinones, iron and copper ions was fitted by KM-SUB-ELF using the Monte Carlo genetic algorithm as detailed below.

Iron and copper were the two transition metal ions included within KM-SUB-ELF. It has been demonstrated experimentally that other transition metals including Mn, Co, V, Ni, Zn, Cd, Cr do not produce H_2O_2 within surrogate lung fluid[3]. Measurements have also shown that there is no production of OH radicals in surrogate lung fluid from any of these metals with the exception of Cu and Fe[4]. Rate constants of Fenton-like reactions do exist for manganese, however, these suggest that the formation of O_2^- (which is the first step of the mechanism) is significantly faster for Fe^{2+} (and hence Cu^{2+}) than for Mn^{2+}[5]. Reactions which interconvert ROS are also significantly faster for Cu and Fe than for Mn[6]. Finally, there is also evidence that total concentrations and water-soluble concentrations of iron and copper within PM2.5 tend to be higher than the concentrations of other water-soluble metal ions such as Mn, Ni, Br, Sr, Pb, As, Ti, Se[7-11].

Note that this study intended to determine a baseline for the primary chemical production of exogenous ROS. From this baseline, air pollutants can cause secondary production or destruction of endogenous ROS via biological interactions and responses of the human immune system, including the activation of macrophages, mitochondria and enzymes like NADPH-oxidase and glutathione peroxidase or infections and microbial growth induced by biological and nutrient-rich particles[12,13]. Activity of enzymes is highly dependent upon exposure to oxidants (e.g. ozone) as well as the pH of the ELF and is significantly reduced in smokers and people suffering from lung diseases such as chronic obstructive pulmonary diseases[14-16].

Supplementary Table 2 summarizes the kinetic parameters including the surface accommodation coefficient, desorption lifetime, Henry's law coefficient, bulk diffusion coefficient, effective molecular cross-section of the gas phase species, mean thermal velocity of the gas phase species and the gas phase diffusion coefficients of O_3 and OH. The concentrations of antioxidants within three different regions of the respiratory tract (the alveoli, the bronchi and the nasal cavity) are known and are summarized within Supplementary Table 3[17]. Albumin-SH has been suggested as an antioxidant but has not been included in the model[18], because it has been demonstrated that it would be of minimal importance compared to GSH[18,19] and there is no evidence of oxidative modification of albumin by ozone in the presence of other antioxidants[20]. The average thickness of the ELF and the diameter of the respiratory tract are also listed. The concentration of the surface lipid was set to 2.2×10^{21} cm^{-3} representing a monolayer of the lipid with a thickness of 7.8×10^{-8} cm equivalent to the effective diameter of the lipid. These values were based on a 60 Å/ lipid surface density[21]. Finally, the surface concentration of reactive sites in surfactant protein was set to 8.8×10^{20} cm^{-3} considering that one surfactant protein molecule contains four reaction sites (the amino acids 2 × cysteine, tryptophan, methionine)[22].

The chemistry of iron and copper with hydrogen peroxide has been extensively studied and can be summarized by the Fenton and ROS reactions shown by Reactions 22 – 48 in Supplementary

Table 1. However, there are large uncertainties in many of these rate coefficients and also in the mechanism. For example, it has been suggested that the reaction of Fe(II) with hydrogen peroxide could lead to the formation of Fe(III) (R26) and/or Fe(IV) (R30) and measurements have suggested that Fe(IV) formation may be more than 1000 times faster at the air-liquid interface than in the bulk[23]. To account for these large uncertainties, the available experimental data sets were reproduced by KM-SUB-ELF. Charrier et al.[3] measured the H_2O_2 production with different iron and copper concentrations in a surrogate ELF solution. Charrier and Anastasio[4] reported the measured para-hydroxybenzoic acid (p-HBA) formed after 24 hours from the reaction of benzoate with OH radicals in surrogate lung fluid as a function of either iron or copper concentration. Surrogate ELF has been widely used and accepted as a suitable surrogate for real ELF, as it contains very similar concentrations of antioxidants and has the same pH of 7.4[3,4,24-27]. We varied the most uncertain kinetic rate constants within their boundaries suggested in the literature. These include oxidation of quinones, Cu(I) and Fe(II) by oxygen species O_2 and $\cdot O_2^-$/ HO_2 (R17, 18, 24, 25, 41, 42, 49), reduction of Cu(II) with $\cdot O_2^-$/ HO_2 (R47 and 48) direct reactions of Cu(I) and Fe(II) with hydrogen peroxide (R26, 30, 43, 44 and 46), the reduction of quinones, Cu(II), Fe(III) and Fe(IV) with ascorbate (R16, 22, 23 and 40) and the reaction of ascorbate with $\cdot O_2^-$ (R51).

To find a common kinetic parameter set describing all available experimental data, the optimization of these rate coefficients was performed by global optimization, which is a genetic algorithm (GA, Matlab Global Optimization Toolbox, Mathworks® software), seeded with results from a uniformly-sampled Monte-Carlo (MC) search for faster convergence (MCGA method)[28,29]. In the MC search, kinetic parameters were varied randomly within their individual bounds. Each data set is fed into the model and the correlation between model output and experimental results is evaluated in a least-squares fashion. In the GA step, a so-called population of parameter sets is optimized by processes resembling recombination and mutation in evolutionary biology. To ensure diversity within the pool of parameter sets and to counteract the sampling bias from shallow local minima, an equal amount of random parameter sets was added to the starting population. The data sets and the fitting obtained with KM-SUB-ELF are shown in Supplementary Fig. 1. Supplementary Figure 11 shows fitted parameters with uncertainty in the box-whisker diagrams based on multiple optimization.

The rate-limiting step for hydrogen peroxide production is found to be the reaction with molecular oxygen with transition metal ions (R24 and 41). For the hydrogen peroxide production by quinones, the radical chain is initiated by oxidation of the antioxidant ascorbate (R13, 16 and 19). Ascorbate also reacts with the oxidized forms of both transition metals (R 22, 23 and 40) and a large portion of hydrogen peroxide production by copper can be attributed to this reaction channel. All reactions involving superoxide and quinone radicals (R17 and 18) were found to be fast, and rate coefficients approach the diffusion limit of $\sim 1\times10^{-11}$ cm^3 s^{-1} [29] (Supplementary Fig. 11).

The main driving force of the production of OH radicals is the direct reactions of hydrogen peroxide with the reduced forms of the trace metals Cu(I) and Fe(II) (R43 and 26). As discussed

above, direct oxidation of Fe(II) to Fe(IV) without production of hydroxyl radicals (R30) is fast in the simulations with a rate of 9.5×10^{-18} cm^3 s^{-1}, which is in line with Enami et al.[23]. Regeneration of the reduced forms of trace metals in the model is mainly accomplished by direct reaction with ascorbate (R22, 23 and 40). Continuous production and destruction of hydrogen peroxide led to a steady-state equilibrium and hence constant production of OH radicals until all of the ascorbate was depleted. It can be seen from the experimental data [4] that the cumulative OH production after 1 day became independent of trace metal concentrations above a certain trace metal concentration (~ 3 – 4 μmol L^{-1}) (Supplementary Fig. 2).

S2. ROS concentrations and OH, H_2O_2 and ROS production rates

To convert ambient concentrations (ng m^{-3}) of transition metal ions, quinones and SOA into ELF concentrations, the following equation was used:

$$ELF\ concentration = \frac{Ambient\ concentration \times Breathing\ rate \times PM\ deposition\ rate \times Fractional\ solubility \times Accumulation\ time}{MW \times Total\ ELF\ volume} \quad (E1)$$

where MW is the molecular weight of the species, the breathing rate was assumed to be 1.5 m^3 h^{-1} [30,31], the PM deposition rate was assumed to be 45% [32] and the total ELF volume was set to 20 ml[33,34]. The fractional solubilities of iron and copper were assumed to be 0.1 and 0.4, respectively[35-37]. Total water-soluble fractions of iron and copper can range from ~5 – 25 % and ~20 – 60 %, respectively, in a wide range of different environments including urban, rural and remote locations[7,35-43], which are represented in Figure 2C by the error bars on each point. Here we assume that species in the particulate matter that undergo reactions are immediately available in solution upon deposition. The temporal evolution of the solvation of different species might also have an influence and should be investigated in future work.

Inhaled particles can be deposited in the respiratory tract and accumulate over several hours before being removed by the immune system and metabolic activity[45,46] and we therefore set the accumulation time to 2 hours in this study. It should also be noted that there is a background concentration of iron within the ELF of a healthy person, but these iron ions are treated as unreactive as they are associated with ferritin and therefore unavailable for Fenton reactions[45,47]. For conversion of typical ambient concentrations into PM2.5 concentrations, it was estimated that 0.13 - 6% of iron, 0.008 - 0.3% of copper, 0.001 - 0.015% of quinones and 12 - 60 % of SOA by weight were present within PM2.5 (Fig. 2A & Supplementary Fig. 5). These values are based upon the measurements shown in Supplementary Tables 4 – 6. For quinones the concentration ratio of [PQN]: [1,2-NQN]: [1,4-NQN] was assumed to be 2: 1: 1.

SOA particles have been shown to contain substantial amounts of ROS, mostly H_2O_2[48-51]. To calculate the ROS concentrations formed in the ELF by SOA, a H_2O_2 formation rate of 1.5×10^9 s^{-1} per microgram of SOA was included in the KM-SUB-ELF model. This value was based on a 0.06 % mass yield of H_2O_2 from terpene SOA at pH 7.5[48]. SOA contains high concentrations of organic

hydroperoxides[52] and recent experiments have shown that SOA can form substantial amounts of OH radicals upon interactions with water and iron ions due to their decomposition[53]. The molar OH yield by SOA was reported to be 0.15% - 1.5% with the highest for β-pinene SOA followed by α-pinene, isoprene, and limonene, whereas naphthalene SOA were found to form negligible OH. We hence assume a 1% molar yield of OH production rate for SOA. It should be noted that these experiments were performed with fresh SOA but some measurements have shown that organic peroxides within SOA would decay on the timescale of about 6 - 20 hours[49,54]; thus, aged SOA might generate lower ROS concentrations. ROS production rates from SOA are equivalent to the sum of the H_2O_2 and OH production rates. Note that the definition of ROS used in our study includes OH, O_2^-, HO_2, O_3, and H_2O_2 in accordance with most biomedical research articles on aerosol lung interactions and health effects[55-57]. However, the broader definition includes organic species such as organic (hydro)peroxides[50,55,58]. Whilst the decomposition of organic hydroperoxides is included within the model, the formation of organic radicals was not included and would be in addition to calculated production rates and concentrations. ROS production rates for iron, copper and quinones were assumed to be equal to O_2^- production rates, as O_2^- is a precursor of all of the other ROS species.

ROS concentrations, shown in Figure 2C, were non-additive due to the coupling of Fe and Cu and their ability to destroy and produce ROS. For example, at a PM2.5 concentration of 400 μg m^{-3} the calculated ROS concentration with iron, copper, SOA and quinones separately and adding these together was up to 3.5 times as high as running the model with all of these PM2.5 components. Due to ROS concentrations being non-additive, an increase of PM2.5 leads to a non-linear increase in ROS concentration due to ROS destruction by Fenton-like reactions for high PM2.5 concentrations in Figure 2C. Nevertheless, ROS concentrations are expected also to be above a critical level in a heavily polluted biomass burning plume in Indonesia. Production rates of ROS, OH and H_2O_2, shown in Figure 2A and Supplementary Figure 5, were additive, within 35 %, 12 % and 15 %, respectively.

Chelating ligands were not specifically considered within the model, as water molecules are mostly acting as ligands in diluted aqueous solutions. Lakey et al.[59] recently demonstrated that several different organics which should act as ligands towards transition metal ions (malonic acid, citric acid, 1,2-diaminoethane, and tartronic acid) did not alter the reactivity of Cu^{2+} with HO_2/ O_2^- which leads to H_2O_2 formation. However, upon addition of oxalic acid a decrease in reactivity of Cu^{2+} with HO_2/ O_2^- was observed, although this only occurred when oxalate was at very high concentrations (10:1 molar ratio of oxalate: copper). Some chelating ligands are also known to increase the rate of H_2O_2 decomposition due to reaction with Fe^{2+}. Addition of halide, sulfate, selenite, trimetaphosphate and tripolyphosphate increased the rate of decomposition of H_2O_2 by Fe^{2+} by a factor of 2 – 3[60]. The fulvic acid complex, and the oxalate complex have both been shown to increase the rate of H_2O_2 decomposition by Fe^{2+} and may also alter the products to form Fe(IV), whereas phosphate ligands can suppress the rate of the reaction[60]. We found that the ROS concentrations may decrease by ~25 % on average if the rate of hydrogen peroxide destruction by Fe^{2+} is increased by a factor of two, for

example if ligands increased the reactivity of the iron. However, there remains uncertainty as to the level of complexing of transition metal ions within the ELF. It should also be noted that there is some variability in the antioxidant concentrations within the ELF; for example, Rahman et al.[44] reported an ascorbate concentration, which is 2.5 times higher than the concentration reported by Mudway and Kelly[103] as shown in Table S3. By using the antioxidant concentrations reported by Rahman et al.[44], an average increase of ~20% would be predicted for the ROS concentrations shown in Fig. 2C due to faster reactions between antioxidants and redox-active components.

In Figure 2D, the effect of removing 50 % of the water soluble fractions of individual or multiple redox-active components upon ROS concentrations within the ELF was investigated. At high PM2.5 concentrations (>~100 μg m^{-3}) the removal of iron will increase ROS concentrations, because iron destroys H_2O_2 by the Fenton reactions (R26, R30) as also observed experimentally in Supplementary Fig. 2A. This is why ROS concentrations saturate or decrease as PM2.5 concentrations increase in Fig. 2C. At lower PM2.5 concentrations, iron contributes more to H_2O_2 production (R24, R25) rather than destruction, and thus the removal of iron decreases ROS concentrations.

Supplementary Figure 4A shows the production rates of OH and H_2O_2 calculated as a function of ambient concentrations of iron, copper, and quinones. With increasing pollutant concentrations, H_2O_2 production rates increase by multiple orders of magnitude from ~10^{-3} pmol L^{-1} s^{-1} under clean conditions up to ~10^{2} pmol L^{-1} s^{-1} at highly polluted conditions. The production rates of OH radicals are several orders of magnitude lower but show an even steeper increase with pollutant concentrations (from ~10^{-9} pmol L^{-1} s^{-1} up to ~10 pmol L^{-1} s^{-1}), in particular with iron and copper ions catalyzing both the formation of H_2O_2 and the conversion of H_2O_2 into OH by Fenton-like reactions. At very high iron and copper concentrations, OH production rates are thus almost as high as H_2O_2 production rates.

As a function of PM2.5 concentration, Supplementary Figure 5 shows H_2O_2 and OH production rate corridors, the range of OH production rates induced by SOA, Fe, Cu, and quinones contained in PM2.5, respectively. The corridors are based on a compilation of the measured components accounting for a certain percentage of the PM2.5 mass in different cities (see Supplementary Tables 4-7). Overall, the H_2O_2 production rate is dominated by Cu and Fe, whereas OH production rate is led by SOA and iron followed by Cu and quinones, mainly reflecting the abundance of each species. Note that recent studies have shown that humic-like substances (HULIS) emitted from biomass burning contain high amounts of quinones that may contribute substantially to ROS production[61-64].

S3. Reactions of oxidants with surfactants and antioxidants

Figure S6 illustrates the temporal evolution of antioxidants, surfactants, and two oxidants of ozone and OH radicals, when ELF in the nasal cavity and the bronchi are exposed to 100 ppb O_3 and 5×10^{6} cm^{-3} OH. The oxidant concentration was kept constant over time, assuming negligible loss of oxidants within the nasal cavity and bronchi over the time period of a single breath of a few seconds.

Fig. S6A shows degradation of total antioxidants by reactions with ozone and OH. Due to the high abundance of antioxidants, antioxidants are depleted only slightly by ~11% in the nasal cavity. In the bronchi the initial antioxidant concentration is higher due to the higher concentration of glutathione. Fig. S6B represents the depletion of surfactants (proteins + lipids) in the different regions of respiratory tract. After 1 hour ~70 % of surfactants were degraded mainly due to reaction with O_3. It should be noted that changing the antioxidant concentrations to those reported by Rahman et al.[44] (as discussed above) had little impact upon the results shown in Fig.S6C and S6D. Inhaled O_3 still rapidly saturates the ELF in all regions of the respiratory tract and inhaled OH is still rapidly scavenged by the surfactants and antioxidants near the surface of the ELF.

Some studies have measured much larger rate coefficients by 2 – 4 orders of magnitude for Reactions 1-4 (although this could be attributed to reactant concentrations as in early studies extremely high O_3 concentrations were used, and the method by which the O_3 contacted the fluid as discussed in [65]). To investigate the effect of these larger rate coefficients upon the temporal evolution of O_3 within the ELF, $k_1 - k_4$ were increased to ~10^{-15} - 10^{-14} cm^3 s^{-1} [66,67]. In the nasal cavity, the O_3 concentration next to the cells and tissues became significantly smaller than the saturation concentration. In the alveoli and bronchi, O_3 was again saturated without any significant concentration gradients despite these higher rate coefficients.

Using the derived uptake coefficients ($\gamma_{O3} = 2 \times 10^{-5}$ and $\gamma_{OH} = 0.96$ in all regions of the respiratory tract) and the surface area of the nasal cavity, bronchi and alveoli (Supplementary Table 3), the number of molecules of O_3 and OH removed in the different regions of the respiratory tract can be estimated. At 100 ppb O_3 and 5×10^6 cm^{-3} OH and assuming that 1 breath of air consists of 2 L, after 1 second O_3 concentrations would decrease by ~0.6% and ~20% in the nasal cavity and bronchi, respectively, whereas OH would decrease by 2% and more than 100% in the nasal cavity and bronchi, respectively. Thus, substantial amounts of O_3 and OH would pass through the nasal cavity and the majority of the loss would occur in the bronchi and alveoli region. This is consistent with estimated chemical half-lives ($t_{1/2}$, the time at which the concentration reached half of the initial concentration) of gas-phase O_3 and OH being on the order of ~10 s in the nasal cavity, ~1 s in bronchi, and ~10 ms in alveoli. Note that turbulent flow within the nasal cavity can occur within a person who is exercising and in this case most of OH radicals would be expected to be lost within the nasal cavity. For ambient OH radicals, $t_{1/2}$ is the longest in the nasal cavity, reflecting that OH loss is mainly limited by gas-phase diffusion.

S4. pH effects on the products

The mean ELF pH of a healthy person has been measured to be ~ 7.4. However, people with diseases such as asthma or acid reflux can experience a decrease in ELF pH down to ~4. The ELF can also be acidified in children or by the inhalation of acidic particles[68,69]. Therefore, it is important to quantify the reactions occurring and the products formed within the ELF at different ELF pH. The

rates of reaction between ozone and the antioxidants are highly dependent upon the pH of the ELF. Enami et al. measured pH dependence of rate coefficients for ozone with ascorbate to be *k*(pH7)/ *k*(pH3) = 2.73[70], the pH dependence for ozone with uric acid was *k*(pH7)/ *k*(pH4) = 380 [71], for ozone reacting with glutathione the dependence was *k*(pH9.2)/ *k*(pH3.5) = 58[73] and finally the dependence for ozone reacting with α-tocopherol was *k*(pH9.3)/ *k*(pH0) = 380[72]. At lower pH, the ratio of products changes with a greater ratio of harmful ozonide, peroxide and epoxide products formed from Reactions 1, 2 and 4. Ozonides are strong oxidizers which can cause significant oxidative damage *in vivo*[74]. In the presence of iron (II), ozonides may also form cytotoxic carbon-centred radicals[75]. Uric acid epoxide (UA-O) could be enzymatically converted to inflammatory 1,2-diols *in vivo*[76].

The pH dependence of the rate coefficients was incorporated into KM-SUB-ELF by assuming that the pH dependence was fully due to the non-protonated antioxidant reacting at a faster rate than the protonated antioxidant. The pKa of the four different antioxidants is known (pKa(Asc) = 4.1, pKa(UA)= 5.4, pKa(GSH) = 8.8 and pKa(α-toc) = 13) and the ratio of the protonated to deprotonated antioxidant could be calculated. Therefore, simultaneous equations can be solved considering the concentration of antioxidant in both the protonated and unprotonated form at two different pHs as well as the change in the rate of reaction between these pHs. The resulting equations are shown below:

$$k_1(\mathrm{pH}) = 0.8625 \times 10^{(\mathrm{pKa(Asc)} - \mathrm{pH})} / (10^{(\mathrm{pKa(Asc)} - \mathrm{pH})} + 1) + 2.7324 \times (1 - 10^{(\mathrm{pKa(Asc)} - \mathrm{pH})} / (10^{(\mathrm{pKa(Asc)} - \mathrm{pH})} + 1)) / 2.7314 \times k_1(\mathrm{pH} = 7.4) \quad \text{(E1)}$$

$$k_2(\mathrm{pH}) = -14.48 \times 10^{(\mathrm{pKa(UA)} - \mathrm{pH})} / (10^{(\mathrm{pKa(UA)} - \mathrm{pH})} + 1) + 389.91 \times (1 - 10^{(\mathrm{pKa\,(UA)} - \mathrm{pH})} / (10^{(\mathrm{pKa\,(UA)} - \mathrm{pH})} + 1)) / 385.91 \times k_2(\mathrm{pH} = 7.4) \quad \text{(E2)}$$

$$k_3(\mathrm{pH}) = 0.9996 \times 10^{(\mathrm{pKa(GSH)} - \mathrm{pH})} / (10^{(\mathrm{pKa\,(GSH)} - \mathrm{pH})} + 1) + 80.692 \times (1 - 10^{(\mathrm{pKa\,(GSH)} - \mathrm{pH})} / (10^{(\mathrm{pKa\,(GSH)} - \mathrm{pH})} + 1)) / 4.051 \times k_3(\mathrm{pH} = 7.4) \quad \text{(E3)}$$

$$k_4(\mathrm{pH}) = (10^{(\mathrm{pKa(\alpha\text{-}Toc)} - \mathrm{pH})} / (10^{(\mathrm{pKa(\alpha\text{-}Toc)} - \mathrm{pH})} + 1) + 1.9 \times 10^{6} \times (1 - 10^{(\mathrm{pKa(\alpha\text{-}Toc)} - \mathrm{pH})} / (10^{(\mathrm{pKa(\alpha\text{-}Toc)} - \mathrm{pH})} + 1)) / 5.772 \times k_4(\mathrm{pH} = 7.4) \quad \text{(E4)}$$

The following equations were incorporated into the model to describe the ascorbate products (DHA, AOZ, THR) ratios [70], and the uric acid epoxide, peroxide and ozonide products (UA-O, UA-O_2, UA-O_3) [71] as a function of pH (Supplementary Fig. 9).

$$[\mathrm{DHA}] = (-0.5786 \times 10^{(\mathrm{pKa(Asc)} - \mathrm{pH})} / (10^{(\mathrm{pKa(Asc)} - \mathrm{pH})} + 1) + 0.8414) \times \text{Asc products} \quad \text{(E5)}$$

$$[\mathrm{AOZ}] = (0.1038 \times 10^{(\mathrm{pKa(Asc)} - \mathrm{pH})} / (10^{(\mathrm{pKa(Asc)} - \mathrm{pH})} + 1) + 0.0086) \times \text{Asc products} \quad \text{(E6)}$$

$$[\mathrm{THR}] = (0.4748 \times 10^{(\mathrm{pKa(Asc)} - \mathrm{pH})} / (10^{(\mathrm{pKa(Asc)} - \mathrm{pH})} + 1) + 0.15) \times \text{Asc products} \quad \text{(E7)}$$

$$[\mathrm{UA\text{-}O}] = (-0.0687 \times 10^{(\mathrm{pKa(UA)} - \mathrm{pH})} / (10^{(\mathrm{pKa(UA)} - \mathrm{pH})} + 1) + 0.9054) \times \text{UA products} \quad \text{(E8)}$$

$$[\mathrm{UA\text{-}O2}] = (0.0051 \times 10^{(\mathrm{pKa(UA)} - \mathrm{pH})} / (10^{(\mathrm{pKa(UA)} - \mathrm{pH})} + 1) + 0.0322) \times \text{UA products} \quad \text{(E9)}$$

$$[\mathrm{UA\text{-}O3}] = (0.0636 \times 10^{(\mathrm{pKa(UA)} - \mathrm{pH})} / (10^{(\mathrm{pKa(UA)} - \mathrm{pH})} + 1) + 0.0614) \times \text{UA products} \quad \text{(E10)}$$

Supplementary Figure 10 shows the expected production of the ascorbate and uric acid ozonation products after 1 hour as a function of pH and the temporal evolution of these products as a function of time and at an ELF pH of 7.4 and 4. Ascorbate ozonide (AOZ) increased from 3.7×10^{13} cm^{-3} to 1.8×10^{14} cm^{-3} from pH 7.4 to pH 4 after 1 hour. Increased oxidative stress of lung cells and tissues in people suffering from pulmonary diseases could be due to such increase formation of AOZ. In contrast, UA-O, UA-O_2 and UA-O_3 are several orders of magnitude smaller at pH 4 compared to pH 7.4.

It should also be noted that Enami et al.[72] previously showed that the presence of α-tocopherol can lead to a decrease in the concentrations of secondary oxidants, such as AOZ and THR. However, the rate coefficients for these reactions remain uncertain and we have therefore not included them within the model. There is also the potential that some of the other secondary oxidants would further react with other antioxidants, although this remains unclear and should be the subject of further investigation. Enami et al.[77] have also previously reported that at low pH the products of the GSH + OH reaction would be sulfenic $GSOH^-$, sulfinic GSO_2^-, and sulfonic GSO_3^- acids rather than GSSG. GSSG is relatively inert and acts a signaling molecule but GSH-sulfenic acid is highly reactive toward oxidants such as ozone[77]. This may have implications for people with an acidic ELF, such as people suffering from asthma.

Lipid ozonation products are a mixture of aldehydes and hydroxyhydroperoxides, which are much more soluble than the initial lipids that may diffuse through the ELF to the lung epithelium[22]. These oxidation products may react with lipases in the lung epithelium releasing endogenous mediators of inflammation[22,78]. Bulk concentrations of lipid ozonation products are estimated to be up to $\sim 1.5 \times 10^{19}$ cm^{-3} (24 mmol L^{-1}) within the alveoli ELF and up to $\sim 7.5 \times 10^{16}$ cm^{-3} (0.1 mmol L^{-1}) within the nasal cavity ELF after one hour exposure of 100 ppb O_3. Note that there are currently no studies of O_3 reactions with surface lipids and proteins over a range of pHs and therefore a change in rate constant and products is currently unknown, which could be important as ozone lipid oxidation products have been shown to be harmful[22,78].

Regarding pH effects on ROS generation by PM2.5, it is known that pH would affect the solubilities of Cu and Fe and also the rate constants of the Fenton and Fenton-like reactions. For example, the rate of reaction R26 will increase by about one order of magnitude between pH 6 and pH 7[79]. However, we do not attempt to investigate the effect of pH upon ROS concentrations by PM2.5 inhalation in this study due to a lack of experimental data for H_2O_2 and OH generation in lung fluid as a function of pH; this aspect should be subject to future experimental and modeling studies.

SI References.

1 Shiraiwa, M., Pfrang, C. & Pöschl, U. Kinetic multi-layer model of aerosol surface and bulk chemistry (KM-SUB): the influence of interfacial transport and bulk diffusion on the oxidation of oleic acid by ozone. *Atmos. Chem. Phys.* **10**, 3673-3691, (2010).

2 Keyhani, K., Scherer, P. W. & Mozell, M. M. Numerical simulation of airflow in the human nasal cavity. *J. Biomech. Eng.* **117**, 429-441, (1995).

3 Charrier, J. G., McFall, A. S., Richards-Henderson, N. K. & Anastasio, C. Hydrogen Peroxide Formation in a Surrogate Lung Fluid by Transition Metals and Quinones Present in Particulate Matter. *Environ. Sci. Technol.* **48**, 7010-7017, (2014).

4 Charrier, J. G. & Anastasio, C. Impacts of antioxidants on hydroxyl radical production from individual and mixed transition metals in a surrogate lung fluid. *Atmos. Environ.* **45**, 7555-7562, (2011).

5 Morgan, J. J. Kinetics of reaction between O_2 and Mn (II) species in aqueous solutions. *Geochim. Cosmochim. Ac.* **69**, 35-48, (2005).

6 Deguillaume, L. *et al.* Transition metals in atmospheric liquid phases: Sources, reactivity, and sensitive parameters. *Chem. Rev.* **105**, 3388-3431, (2005).

7 Fang, T., Guo, H., Verma, V., Peltier, R. E. & Weber, R. J. PM2.5 water-soluble elements in the southeastern United States: automated analytical method development, spatiotemporal distributions, source apportionment, and implications for heath studies. *Atmos. Chem. Phys.* **15**, 11667-11682, (2015).

8 Harrison, R. M. & Yin, J. Chemical speciation of PM2.5 particles at urban background and rural sites in the UK atmosphere. *J. Environ. Monitor.* **12**, 1404-1414, (2010).

9 Squizzato, S. *et al.* The PM2.5 chemical composition in an industrial zone included in a large urban settlement: main sources and local background. *Env. Sci. Pro. Imp.* **16**, 1913-1922, (2014).

10 Xiao, Y.-H. *et al.* Characteristics and Sources of Metals in TSP and PM2.5 in an Urban Forest Park at Guangzhou. *Atmosphere* **5**, 775-787, (2014).

11 Contini, D., Cesari, D., Donateo, A., Chirizzi, D. & Belosi, F. Characterization of PM10 and PM2.5 and Their Metals Content in Different Typologies of Sites in South-Eastern Italy. *Atmosphere* **5**, 435-453, (2014).

12 Borcherding, J. *et al.* Iron oxide nanoparticles induce Pseudomonas aeruginosa growth, induce biofilm formation, and inhibit antimicrobial peptide function. *Environ. Sci. Nano* **1**, 123-132, (2014).

13 Stohs, S. & Bagchi, D. Oxidative mechanisms in the toxicity of metal ions. *Free Radical Bio. Med.* **18**, 321-336, (1995).

14 Pannala, V., Bazil, J., Camara, A. & Dash, R. A mechanistic mathematical model for the catalytic action of glutathione peroxidase. *Free Radical Res.* **48**, 487-502, (2014).

15 Bentley, A. R., Emrani, P. & Cassano, P. A. Genetic variation and gene expression in antioxidant-related enzymes and risk of chronic obstructive pulmonary disease: a systematic review. *Thorax* **63**, 956-961, (2008).

16 Avissar, N. E., Reed, C. K., Cox, C., Frampton, M. W. & Finkelstein, J. N. Ozone, but not nitrogen dioxide, exposure decreases glutathione peroxidases in epithelial lining fluid of human lung. *Am. J. Respir. Crit. Care Med.* **162**, 1342-1347, (2000).

17 van der Vliet, A. *et al.* Determination of low-molecular-mass antioxidant concentrations in human respiratory tract lining fluids. *Am. J. Physiol.- Lung C.* **276**, L289-L296, (1999).

18 Cross, C. E., van der Vliet, A., Louie, S., Thiele, J. J. & Halliwell, B. Oxidative stress and antioxidants at biosurfaces: Plants, skin, and respiratory tract surfaces. *Environ. Health Perspect.* **106**, 1241-1251, (1998).

19 Cross, C. E., van der Vliet, A., O'Neill, C. A., Louie, S. & Halliwell, B. Oxidants, antioxidants, and respiratory tract lining fluids. *Environ. Health Perspect.* **102**, 185, (1994).

20 Mudway, I. S. & Kelly, F. J. Modeling the interactions of ozone with pulmonary epithelial lining fluid antioxidants. *Toxicol. Appl. Pharm.* **148**, 91-100, (1998).

21 Kim, H. I. *et al.* Interfacial Reactions of Ozone with Surfactant Protein B in a Model Lung Surfactant System. *J. Am. Chem. Soc.* **132**, 2254-2263, (2010).

22 Pryor, W. A., Squadrito, G. L. & Friedman, M. The Cascade Mechanism to Explain Ozone Toxicity - The Role of Lipid Ozonation Products. *Free Radical Bio. Med.* **19**, 935-941, (1995).
23 Enami, S., Sakamoto, Y. & Colussi, A. J. Fenton chemistry at aqueous interfaces. *P. Natl. Acad. Sci. U.S.A* **111**, 623-628, (2014).
24 Davies, N. M. & Feddah, M. R. A novel method for assessing dissolution of aerosol inhaler products. *Int. J. Pharm.* **255**, 175-187, (2003).
25 Jung, H., Guo, B., Anastasio, C. & Kennedy, I. M. Quantitative measurements of the generation of hydroxyl radicals by soot particles in a surrogate lung fluid. *Atmos. Environ.* **40**, 1043-1052, (2006).
26 Vidrio, E., Phuah, C. H., Dillner, A. M. & Anastasio, C. Generation of Hydroxyl Radicals from Ambient Fine Particles in a Surrogate Lung Fluid Solution. *Environ. Sci. Tech.* **43**, 922-927, (2009).
27 Moss, O. R. Simulants Of Lung Interstitial Fluid. *Health Phys.* **36**, 447-448, (1979).
28 Berkemeier, T. *et al.* Kinetic regimes and limiting cases of gas uptake and heterogeneous reactions in atmospheric aerosols and clouds: a general classification scheme. *Atmos. Chem. Phys.* **13**, 6663-6686, (2013).
29 Arangio, A. M. *et al.* Multiphase Chemical Kinetics of OH Radical Uptake by Molecular Organic Markers of Biomass Burning Aerosols: Humidity and Temperature Dependence, Surface Reaction and Bulk Diffusion. *J. Phys. Chem. A* **119**, 4533–4544, (2015).
30 Spier, C. *et al.* Activity patterns in elementary and high school students exposed to oxidant pollution. *J. Expo. Anal. Env. Epid.* **2**, 277-293, (1991).
31 Agency, U. S. E. P. *Exposure Factors Handbook*. Vol. EPA/600/R-09/052F (2011).
32 Sarangapani, R. & Wexler, A. S. The role of dispersion in particle deposition in human airways. *Toxicol. Sci.* **54**, 229-236, (2000).
33 Rennard, S. *et al.* Estimation of volume of epithelial lining fluid recovered by lavage using urea as marker of dilution. *J. Appl. Physiol.* **60**, 532-538, (1986).
34 Walters, D. V. Lung lining liquid–The hidden depths. *Neonatology* **81**, 2-5, (2002).
35 Connell, D. P., Winter, S. E., Conrad, V. B., Kim, M. & Crist, K. C. The Steubenville Comprehensive Air Monitoring Program (SCAMP): Concentrations and solubilities of PM2.5 trace elements and their implications for source apportionment and health research. *J. Air Waste Manage.* **56**, 1750-1766, (2006).
36 Manousakas, M., Papaefthymiou, H., Eleftheriadis, K. & Katsanou, K. Determination of water-soluble and insoluble elements in PM2.5 by ICP-MS. *Sci. Total Environ.* **493**, 694-700, (2014).
37 Heal, M. R., Hibbs, L. R., Agius, R. M. & Beverland, L. J. Total and water-soluble trace metal content of urban background PM10, PM2.5 and black smoke in Edinburgh, UK. *Atmos. Environ.* **39**, 1417-1430, (2005).
38 Birmili, W., Allen, A. G., Bary, F. & Harrison, R. M. Trace metal concentrations and water solubility in size-fractionated atmospheric particles and influence of road traffic. *Environ. Sci. Tech.* **40**, 1144-1153, (2006).
39 Espinosa, A. J. F., Rodríguez, M. T., de la Rosa, F. J. B. & Sánchez, J. C. J. A chemical speciation of trace metals for fine urban particles. *Atmos. Environ.* **36**, 773-780, (2002).
40 Baker, A., Jickells, T., Witt, M. & Linge, K. Trends in the solubility of iron, aluminium, manganese and phosphorus in aerosol collected over the Atlantic Ocean. *Mar. Chem.* **98**, 43-58, (2006).
41 Buck, C. S., Landing, W. M., Resing, J. A. & Measures, C. I. The solubility and deposition of aerosol Fe and other trace elements in the north atlantic ocean: observations from the A16N CLIVAR/CO 2 repeat hydrography section. *Mar. Chem.* **120**, 57-70, (2010).
42 Oakes, M., Weber, R., Lai, B., Russell, A. & Ingall, E. Characterization of iron speciation in urban and rural single particles using XANES spectroscopy and micro X-ray fluorescence measurements: investigating the relationship between speciation and fractional iron solubility. *Atmos. Chem. Phys.* **12**, 745-756, (2012).
43 Jiang, S. Y., Yang, F., Chan, K. L. & Ning, Z. Water solubility of metals in coarse PM and PM 2.5 in typical urban environment in Hong Kong. *Atmos. Poll. Res.* **5**, 236-244, (2014).

44 Rahman, I., Biswas, S. K. & Kode, A. Oxidant and antioxidant balance in the airways and airway diseases. *European journal of pharmacology* **533**, 222-239, (2006).
45 Ghio, A. J., Turi, J. L., Yang, F., Garrick, L. M. & Garrick, M. D. Iron homeostasis in the lung. *Biol. Res.* **39**, 67-77, (2006).
46 Ghio, A. J., Richards, J. H., Dittrich, K. L. & Samet, J. M. Metal storage and transport proteins increase after exposure of the rat lung to an air pollution particle. *Toxicol. Path.* **26**, 388-394, (1998).
47 Ghio, A. J. Disruption of iron homeostasis and lung disease. *B. B. A. - Gen. Subjects* **1790**, 731-739, (2009).
48 Wang, Y., Kim, H. & Paulson, S. E. Hydrogen peroxide generation from alpha- and beta-pinene and toluene secondary organic aerosols. *Atmos. Environ.* **45**, 3149-3156, (2011).
49 Chen, X., Hopke, P. K. & Carter, W. P. L. Secondary Organic Aerosol from Ozonolysis of Biogenic Volatile Organic Compounds: Chamber Studies of Particle and Reactive Oxygen Species Formation. *Environ. Sci. Tech.* **45**, 276-282, (2011).
50 Hopke, P. K. *Chapter 1 in Air Pollution and Health Effects*. (Springer, 2015).
51 Wang, Y., Arellanes, C. & Paulson, S. E. Hydrogen Peroxide Associated with Ambient Fine-Mode, Diesel, and Biodiesel Aerosol Particles in Southern California. *Aerosol Sci. Tech.* **46**, 394-402, (2012).
52 Docherty, K. S., Wu, W., Lim, Y. B. & Ziemann, P. J. Contributions of organic peroxides to secondary aerosol formed from reactions of monoterpenes with O_3. *Environ. Sci. Tech.* **39**, 4049-4059, (2005).
53 Tong, H. *et al.* Hydroxyl radicals from secondary organic aerosol decomposition in water. *Atmos. Chem. Phys.* **16**, 1761-1771, (2016).
54 Badali, K. M. *et al.* Formation of hydroxyl radicals from photolysis of secondary organic aerosol material. *Atmos. Chem. Phys.* **15**, 7831-7840, (2015).
55 Pöschl, U. & Shiraiwa, M. Multiphase Chemistry at the Atmosphere–Biosphere Interface Influencing Climate and Public Health in the Anthropocene. *Chem. Rev.* **115**, 4440–4475, (2015).
56 Winterbourn, C. C. Reconciling the chemistry and biology of reactive oxygen species. *Nature Chem. Biol.* **4**, 278-286, (2008).
57 Janssen, N. A. H., VanMansom, D. F. M., VanDerJagt, K., Harssema, H. & Hoek, G. Mass concentration and elemental composition of airborne particulate matter at street and background locations. *Atmos. Environ.* **31**, 1185-1193, (1997).
58 Shiraiwa, M. *et al.* The role of long-lived reactive oxygen intermediates in the reaction of ozone with aerosol particles. *Nature Chem.* **3**, 291-295, (2011).
59 Lakey, P. S. J., George, I. J., Baeza-Romero, M. T., Whalley, L. K. & Heard, D. E. Organics substantially reduce HO_2 uptake onto aerosols containing transition metal ions. *J. Phys. Chem. A* **120**, 1421–1430, (2015).
60 Pignatello, J. J., Oliveros, E. & MacKay, A. Advanced oxidation processes for organic contaminant destruction based on the Fenton reaction and related chemistry. *Crit. Rev. Env. Sci. Tech.* **36**, 1-84, (2006).
61 Lin, P. & Yu, J. Z. Generation of Reactive Oxygen Species Mediated by Humic-like Substances in Atmospheric Aerosols. *Environ. Sci. Tech.* **45**, 10362-10368, (2011).
62 Verma, V. *et al.* Organic Aerosols Associated with the Generation of Reactive Oxygen Species (ROS) by Water-Soluble PM2.5. *Environ. Sci. Technol.* **49**, 4646-4656, (2015).
63 Verma, V. *et al.* Fractionating ambient humic-like substances (HULIS) for their reactive oxygen species activity – Assessing the importance of quinones and atmospheric aging. *Atmos. Environ.* **120**, 351-359, (2015).
64 Dou, J., Lin, P., Kuang, B.-Y. & Yu, J. Z. Reactive Oxygen Species Production Mediated by Humic-like Substances in Atmospheric Aerosols: Enhancement Effects by Pyridine, Imidazole, and Their Derivatives. *Environ. Sci. Technol.* **49**, 6457-6465, (2015).
65 Kermani, S., Ben-Jebria, A. & Ultman, J. S. Kinetics of ozone reaction with uric acid, ascorbic acid, and glutathione at physiologically relevant conditions. *Arch. Biochem. Biophys.* **451**, 8-16, (2006).

66 Giamalva, D., Church, D. F. & Pryor, W. A. A Comparison of the Rates Of Ozonation of Biological Antioxidants and Oleate and Linoleate Esters. *Bio. Chem. Bioph. Res. Co.* **133**, 773-779, (1985).

67 Kanofsky, J. R. & Sima, P. D. Reactive Absorption of Ozone by Aqueous Biomolecule Solutions - Implications for the Role of Sulfhydryl Compounds as Targets for Ozone. *Arch. Biochem. Biophys.* **316**, 52-62, (1995).

68 Paget-Brown, A. O. *et al.* Normative data for pH of exhaled breath condensate. *Chest* **129**, 426-430, (2006).

69 Ricciardolo, F. L. M., Gaston, B. & Hunt, J. Acid stress in the pathology of asthma. *J. Allergy Clin. Immun.* **113**, 610-619, (2004).

70 Enami, S., Hoffmann, M. R. & Colussi, A. J. Acidity enhances the formation of a persistent ozonide at aqueous ascorbate/ozone gas interfaces. *P. Natl. Acad. Sci. U.S.A* **105**, 7365-7369, (2008).

71 Enami, S., Hoffmann, M. R. & Colussi, A. J. Ozonolysis of uric acid at the air/water interface. *J. Phys. Chem. B* **112**, 4153-4156, (2008).

72 Enami, S., Hoffmann, M. R. & Colussi, A. J. How Phenol and α-Tocopherol React with Ambient Ozone at Gas/Liquid Interfaces. *J. Phys. Chem. A* **113**, 7002-7010, (2009).

73 Enami, S., Hoffmann, M. R. & Colussi, A. J. Ozone Oxidizes Glutathione to a Sulfonic Acid. *Chem. Res. Toxicol.* **22**, 35-40, (2009).

74 Tang, Y. *et al.* Weak base dispiro-1,2,4-trioxolanes: Potent antimalarial ozonides. *Bioorg. Med. Chem. Lett.* **17**, 1260-1265, (2007).

75 Mercer, A. E. *et al.* Evidence for the involvement of carbon-centered radicals in the induction of apoptotic cell death by artemisinin compounds. *J. Biol. Chem.* **282**, 9372-9382, (2007).

76 Smith, K. R. *et al.* Attenuation of tobacco smoke-induced lung inflammation by treatment with a soluble epoxide hydrolase inhibitor. *P. Natl. Acad. Sci. U.S.A* **102**, 2186-2191, (2005).

77 Enami, S., Hoffmann, M. R. & Colussi, A. J. OH-Radical Specific Addition to Glutathione S-Atom at the Air–Water Interface: Relevance to the Redox Balance of the Lung Epithelial Lining Fluid. *The Journal of Physical Chemistry Letters* **6**, 3935-3943, (2015).

78 Salgo, M. G., Squadrito, G. L. & Pryor, W. A. Activation of phospholipase A_2 in 1-palmitoyl-2-oleoyl-sn-Glycero-3-phosphocholine liposomes containing lipid ozonation products. *Chem. Res. Toxicol.* **7**, 458-462, (1994).

79 Bataineh, H., Pestovsky, O. & Bakac, A. pH-induced mechanistic changeover from hydroxyl radicals to iron(IV) in the Fenton reaction. *Chem. Sci.* **3**, 1594-1599, (2012).

80 Pryor, W. A., Giamalva, D. H. & Church, D. F. Kinetics of Ozonation .2. Amino-Acids and Model Compounds in Water and Comparisons to Rates in Nonpolar-Solvents. *J. Am. Chem. Soc.* **106**, 7094-7100, (1984).

81 Adams, G. E., Boag, J. W., Currant, J. & Michael, B. D. *Absolute rate constants for the reaction of the hydroxyl radical with organic compounds*. (1965).

82 Masuda, T., Shinohara, H. & Kondo, M. Reactions of hydroxyl radicals with nucleic-acid bases and the related compounds in gamma irradiated aqueous solution. *J. Radiat. Res.* **16**, 153-161, (1975).

83 Eriksen, T. E. & Fransson, G. Formation of reducing radicals on radiolysis of glutathione and some related-compounds in aqueous-solution. *J. Chem. Soc. Perk. T. 2*, 1117-1122, (1988).

84 Liphard, M., Bothe, E. & Schultefrohlinde, D. The influence of glutathione on single-strand breakage in single-stranded-DNA irradiated in aqueous-solution in the absence and presence of oxygen. *Int. J. Radiat. Bio.* **58**, 589-602, (1990).

85 Navarrete, M., Rangel, C., Corchado, J. C. & Espinosa-García, J. Trapping of the OH Radical by α-Tocopherol: A Theoretical Study. *J. Phys. Chem. A* **109**, 4777-4784, (2005).

86 Buxton, G. V., Greenstock, C. L., Helman, W. P. & Ross, A. B. Critical-review of rate constants for reactions of hydrated electrons, hydrogen-atoms and hydroxyl radicals ($\bullet OH/\bullet O^-$) in aqueous-solution. *J. Phys. Chem. Ref. Data* **17**, 513-886, (1988).

87 Hoffman, M. Z. & Hayon, E. Pulse-radiolysis study of sulfhydryl compounds in aqueous-solution. *J. Phys. Chem. - U. S.* **77**, 990-996, (1973).

88 Zaho, M. J., Jung, L., Tanielian, C. & Mechin, R. Kinetics of the competitive degradation of deoxyribose and other biomolecules by hydroxyl radicals produced by the Fenton reaction. *Free Radical Res.* **20**, 345-363, (1994).

89 Stuglik, Z. & Zagorski, Z. P. Pulse-radiolysis of neutral iron(II) solutions - Oxidation of ferrous ions by OH radicals. *Radiat. Phys. Chem.* **17**, 229-233, (1981).

90 Lewis, S. *et al.* Chelate-Modified Fenton Reaction for the Degradation of Trichloroethylene in Aqueous and Two-Phase Systems. *Env. Eng. Sci.* **26**, 849-859, (2009).

91 Rush, J. D. & Bielski, B. H. J. Pulse radiolytic studies of the reactions of HO_2/O_2^- with Fe(II)/Fe(III) ions - The reactivity of HO_2/O_2^- with ferric ions and its implication on the occurence of the Haber-Weiss reaction. *J. Phys. Chem. - U. S.* **89**, 5062-5066, (1985).

92 Hug, S. J. & Leupin, O. Iron-catalyzed oxidation of arsenic(III) by oxygen and by hydrogen peroxide: pH-dependent formation of oxidants in the Fenton reaction. *Environ. Sci. Tech.* **37**, 2734-2742, (2003).

93 Christensen, H., Sehested, K. & Corfitzen, H. Reactions of hydroxyl radicals with hydrogen peroxide at ambient and elevated temperatures. *J. Phys. Chem. - U. S.* **86**, 1588-1590, (1982).

94 Sehested, K., Rasmusse.Ol & Fricke, H. Rate constants of OH with HO_2, O_2^- and $H_2O_2^+$ from hydrogen peroxide formation in pulse irradiated oxygenated water. *J. Phys. Chem. - U. S.* **72**, 626-&, (1968).

95 Koppenol, W. H., Butler, J. & Vanleeuwen, J. W. The Haber-Weiss cycle. *Photochem. Photobiol.* **28**, 655-660, (1978).

96 Thornton, J. A., Jaeglé, L. & McNeill, V. F. Assessing known pathways for HO_2 loss in aqueous atmospheric aerosols: Regional and global impacts on tropospheric oxidants. *J. Geophys. Res. - Atmos.* **113**, (2008).

97 Divišek, J. & Kastening, B. Electrochemical generation and reactivity of the superoxide ion in aqueous solutions. *J. Electroanal. Chem. Interface Electrochem.* **65**, 603-621, (1975).

98 Ulanski, P. & von Sonntag, C. Stability constants and decay of aqua-copper(III) - A study by pulse radiolysis with conductometric and optical detection. *Eur. J. Inorg. Chem.*, 1211-1217, (2000).

99 Packer, L. *Vitamin C in health and disease*. Vol. 4 (CRC Press, 1997).

100 Buxton, G. V., Greenstock, C. L., Helman, W. P. & Ross, A. B. Critical review of rate constants for reactions of hydrated electrons, hydrogen atoms and hydroxyl radicals (· OH/· O^- in aqueous solution. *J. Phys. Chem. Ref. Data* **17**, 513-886, (1988).

101 Kohen, R. & Nyska, A. Oxidation of biological systems: Oxidative stress phenomena, antioxidants, redox reactions, and methods for their quantification. *Toxicol. Pathol.* **30**, 620-650, (2002).

102 Sander, R. Compilation of Henry's law constants (version 4.0) for water as solvent. *Atmos. Chem. Phys.* **15**, 4399-4981, (2015).

103 Mudway, I. S. & Kelly, F. J. Ozone and the lung: A sensitive issue. *Mol. Aspects Med.* **21**, 1-48, (2000).

104 Ochs, M. *et al.* The number of alveoli in the human lung. *Am. J. Respir. Crit. Care Med.* **169**, 120-124, (2004).

105 Franciscus, R. G. & Long, J. C. Variation in human nasal height and breadth. *A. J. Phys. Anthropol.* **85**, 419-427, (1991).

106 Arana, A. A., Artaxo, P., Rizzo, L. V. & Bastos, W. Long term measurements of the elemental composition and optical properties of aerosols in Amazonia. *Proceedings of the 16th International Conference on Heavy Metals in the Environment* **1**, 03005, (2013).

107 Maenhaut, W., Salma, I., Cafmeyer, J., Annegarn, H. J. & Andreae, M. O. Regional atmospheric aerosol composition and sources in the eastern Transvaal, South Africa, and impact of biomass burning. *J. Geophys. Res. - Atmos.* **101**, 23631-23650, (1996).

108 Artaxo, P., Gerab, F., Yamasoe, M. A. & Martins, J. V. Fine mode aerosol composition at three long - term atmospheric monitoring sites in the Amazon Basin. *J. Geophys. Res. - Atmos.* **99**, 22857-22868, (1994).

109 Pakkanen, T. A. *et al.* Sources and chemical composition of atmospheric fine and coarse particles in the Helsinki area. *Atmos. Environ.* **35**, 5381-5391, (2001).

110 Olson, D. A. *et al.* Indoor and outdoor concentrations of organic and inorganic molecular markers: Source apportionment of PM2.5 using low-volume samples. *Atmos. Environ.* **42**, 1742-1751, (2008).

111 Lee, P. K. H., Brook, J. R., Dabek-Zlotorzynska, E. & Mabury, S. A. Identification of the major sources contributing to PM2.5 observed in Toronto. *Environ. Sci. Tech.* **37**, 4831-4840, (2003).

112 Upadhyay, N., Clements, A., Fraser, M. & Herckes, P. Chemical Speciation of PM2.5 and PM10 in South Phoenix, AZ. *J. Air Waste Manage.* **61**, 302-310, (2011).

113 Hassanvand, M. S. *et al.* Characterization of PAHs and metals in indoor/outdoor PM10/PM2.5/PM1 in a retirement home and a school dormitory. *Sci. Total Environ.* **527**, 100-110, (2015).

114 Hidemori, T. *et al.* Characteristics of atmospheric aerosols containing heavy metals measured on Fukue Island, Japan. *Atmos. Environ.* **97**, 447-455, (2014).

115 Han, Y.-J., Kim, H.-W., Cho, S.-H., Kim, P.-R. & Kim, W.-J. Metallic elements in PM2.5 in different functional areas of Korea: Concentrations and source identification. *Atmos. Res.* **153**, 416-428, (2015).

116 Maenhaut, W. *et al.* Chemical composition and mass closure for fine and coarse aerosols at a kerbside in Budapest, Hungary, in spring 2002. *X-Ray Spectrom.* **34**, 290-296, (2005).

117 Rogula-Kozlowska, W. *et al.* PM2.5 in the central part of Upper Silesia, Poland: concentrations, elemental composition, and mobility of components. *Environ. Monit. Assess.* **185**, 581-601, (2013).

118 Morishita, M. *et al.* Identification of ambient PM2.5 sources and analysis of pollution episodes in Detroit, Michigan using highly time-resolved measurements. *Atmos. Environ.* **45**, 1627-1637, (2011).

119 Chow, J. C. *et al.* Temporal and spatial variations pf PM2.5 and PM10 aerosol in the southern California air-quality study. *Atmos. Environ.* **28**, 2061-2080, (1994).

120 Vecchi, R., Marcazzan, G., Valli, G., Ceriani, M. & Antoniazzi, C. The role of atmospheric dispersion in the seasonal variation of PM1 and PM2.5 concentration and composition in the urban area of Milan (Italy). *Atmos. Environ.* **38**, 4437-4446, (2004).

121 Khodeir, M. *et al.* Source apportionment and elemental composition of PM2.5 and PM10 in Jeddah City, Saudi Arabia. *Atmos. Poll. Res.* **3**, 331-340, (2012).

122 Hagler, G. S. W. *et al.* Local and regional anthropogenic influence on PM2.5 elements in Hong Kong. *Atmos. Environ.* **41**, 5994-6004, (2007).

123 Loyola, J., Arbilla, G., Quiterio, S. L., Escaleira, V. & Minho, A. S. Trace Metals in the Urban Aerosols of Rio de Janeiro City. *J. Brazil Chem. Soc.* **23**, 628-638, (2012).

124 Matschullat, J., Maenhaut, W., Zimmermann, F. & Fiebig, J. Aerosol and bulk deposition trends in the 1990's, Eastern Erzgebirge, Central Europe. *Atmos. Environ.* **34**, 3213-3221, (2000).

125 Querol, X. *et al.* PM 10 and PM2.5 source apportionment in the Barcelona Metropolitan area, Catalonia, Spain. *Atmos. Environ.* **35**, 6407-6419, (2001).

126 Martinez, M. A., Caballero, P., Carrillo, O., Mendoza, A. & Manuel Mejia, G. Chemical characterization and factor analysis of PM2.5 in two sites of Monterrey, Mexico. *J. Air Waste Manage.* **62**, 817-827, (2012).

127 Na, K. & Cocker, D. R., III. Characterization and source identification of trace elements in PM2.5 from Mira Loma, Southern California. *Atmos. Res.* **93**, 793-800, (2009).

128 Shaltout, A. A., Boman, J., Al-Malawi, D.-a. R. & Shehadeh, Z. F. Elemental Composition of PM2.5 Particles Sampled in Industrial and Residential Areas of Taif, Saudi Arabia. *Aerosol Air Qual. Res.* **13**, 1356-1364, (2013).

129 Chow, J. C. *et al.* Descriptive analysis of PM 2.5 and PM 10 at regionally representative locations during SJVAQS/AUSPEX. *Atmos. Environ.* **30**, 2079-2112, (1996).

130 Kendall, M., Pala, K., Ucakli, S. & Gucer, S. Airborne particulate matter (PM2.5 and PM10) and associated metals in urban Turkey. *Air Qual. Atmos. Health* **4**, 235-242, (2011).

131 Mansha, M., Ghauri, B., Rahman, S. & Amman, A. Characterization and source apportionment of ambient air particulate matter (PM2.5) in Karachi. *Sci. Total Environ.* **425**, 176-183, (2012).

132 Pant, P. *et al.* Characterization of ambient PM2.5 at a pollution hotspot in New Delhi, India and inference of sources. *Atmos. Environ.* **109**, 178-189, (2015).

133 Tolis, E. I. *et al.* One-year intensive characterization on PM2.5 nearby port area of Thessaloniki, Greece. *Environ. Sci. Pollut. R.* **22**, 6812-6826, (2015).

134 Laura Lopez, M. *et al.* Elemental concentration and source identification of PM10 and PM2.5 by SR-XRF in Cordoba City, Argentina. *Atmos. Environ.* **45**, 5450-5457, (2011).

135 Cao, L., Zeng, J., Liu, K., Bao, L. & Li, Y. Characterization and Cytotoxicity of PM<0.2, PM0.2-2.5 and PM2.5-10 around MSWI in Shanghai, China. *Int. J. Environ. Res. Pub. Health* **12**, 5076-5089, (2015).

136 Yin, L. *et al.* Chemical compositions of PM2.5 aerosol during haze periods in the mountainous city of Yong'an, China. *J. Environ. Sci. - China* **24**, 1225-1233, (2012).

137 Zhou, S. *et al.* Trace metals in atmospheric fine particles in one industrial urban city: Spatial variations, sources, and health implications. *J. Environ. Sci. - China* **26**, 205-213, (2014).

138 Wang, X., Bi, X., Sheng, G. & Fu, J. Chemical composition and sources of PM10 and PM2.5 aerosols in Guangzhou, China. *Environ. Monit. Assess.* **119**, 425-439, (2006).

139 Kulshrestha, A., Satsangi, P. G., Masih, J. & Taneja, A. Metal concentration of PM2.5 and PM10 particles and seasonal variations in urban and rural environment of Agra, India. *Sci. Total Environ.* **407**, 6196-6204, (2009).

140 Yadav, S. & Satsangi, P. G. Characterization of particulate matter and its related metal toxicity in an urban location in South West India. *Environ. Monit. Assess.* **185**, 7365-7379, (2013).

141 Song, S. *et al.* Chemical characteristics of size-resolved PM2.5 at a roadside environment in Beijing, China. *Environ. Pollut.* **161**, 215-221, (2012).

142 Sun, Y. L. *et al.* The air-borne particulate pollution in Beijing - concentration, composition, distribution and sources. *Atmos. Environ.* **38**, 5991-6004, (2004).

143 See, S. W., Balasubramanian, R., Rianawati, E., Karthikeyan, S. & Streets, D. G. Characterization and source apportionment of particulate matter $\leq$ 2.5 μm in Sumatra, Indonesia, during a recent peat fire episode. *Environ. Sci. Tech.* **41**, 3488-3494, (2007).

144 Wingfors, H., Hagglund, L. & Magnusson, R. Characterization of the size-distribution of aerosols and particle-bound content of oxygenated PAHs, PAHs, and n-alkanes in urban environments in Afghanistan. *Atmos. Environ.* **45**, 4360-4369, (2011).

145 Valavanidis, A., Fiotakis, K., Vlahogianni, T., Papadimitriou, V. & Pantikaki, V. Determination of selective quinones and quinoid radicals in airborne particulate matter and vehicular exhaust particles. *Environ. Chem.* **3**, 118-123, (2006).

146 Eiguren-Fernandez, A. *et al.* Atmospheric distribution of gas- and particle-phase quinones in Southern California. *Aerosol Sci. Tech.* **42**, 854-861, (2008).

147 Delgado-Saborit, J. M., Alam, M. S., Pollitt, K. J. G., Stark, C. & Harrison, R. M. Analysis of atmospheric concentrations of quinones and polycyclic aromatic hydrocarbons in vapour and particulate phases. *Atmos. Environ.* **77**, 974-982, (2013).

148 Walgraeve, C. *et al.* Quantification of PAHs and oxy-PAHs on airborne particulate matter in Chiang Mai, Thailand, using gas chromatography high resolution mass spectrometry. *Atmos. Environ.* **107**, 262-272, (2015).

149 Alam, M. S., Delgado-Saborit, J. M., Stark, C. & Harrison, R. M. Investigating PAH relative reactivity using congener profiles, quinone measurements and back trajectories. *Atmos. Chem. Phys.* **14**, 2467-2477, (2014).

150 Cho, A. K. *et al.* Determination of four quinones in diesel exhaust particles, SRM 1649a, an atmospheric PM2.5. *Aerosol Sci. Tech.* **38**, 68-81, (2004).

151 Sousa, E. T., Cardoso, M. P., Silva, L. A. & de Andrade, J. B. Direct determination of quinones in fine atmospheric particulate matter by GC-MS. *Microchem. J.* **118**, 26-31, (2015).

152 Allen, J. O. *et al.* Measurement of oxygenated polycyclic aromatic hydrocarbons associated with a size-segregated urban aerosol. *Environ. Sci. Tech.* **31**, 2064-2070, (1997).

153 Jimenez, J. L. *et al.* Evolution of Organic Aerosols in the Atmosphere. *Science* **326**, 1525-1529, (2009).

154 Huang, R.-J. *et al.* High secondary aerosol contribution to particulate pollution during haze events in China. *Nature* **514**, 218-222, (2014).

155 Pöschl, U. *et al.* Rainforest Aerosols as Biogenic Nuclei of Clouds and Precipitation in the Amazon. *Science* **329**, 1513-1516, (2010).

Figures

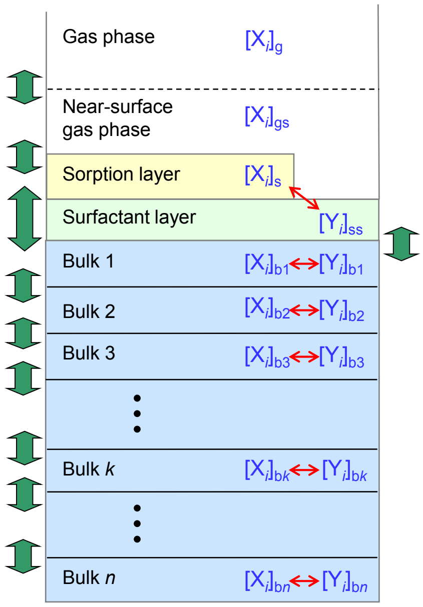

Supplementary Figure 1: A schematic of the KM-SUB-ELF model. The symbols X_i and Y_i represent volatile and non-volatile species, respectively. Green and red arrows denote mass transport and chemical reactions, respectively. Adapted from Shiraiwa et al.[1].

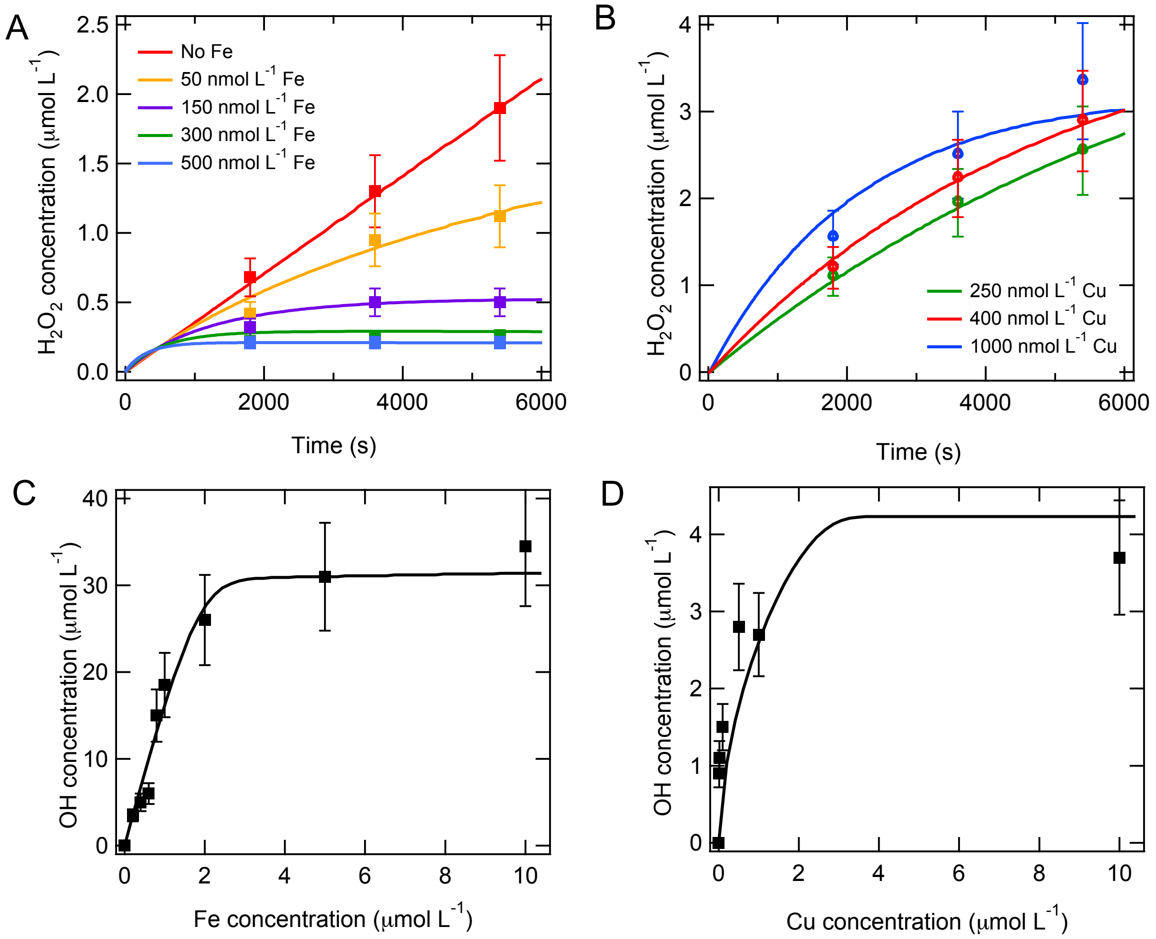

Supplementary Figure 2: Reactive oxygen species (ROS) concentrations in surrogate epithelial lining fluid. H_2O_2 concentration as a function of time at different concentrations of Fe ions (A) and Cu ions (B). OH radical concentration after 24 hours as a function of Fe ion concentration (C) and Cu ion concentration (D). The symbols are measurement data points (for H_2O_2[3] and for OH[4]), and the lines are KM-SUB-ELF model results. In panel (A) the fluid contained 20 nM 1,2-NQN. The error bars represent assumed 20 % errors in the measurement points.

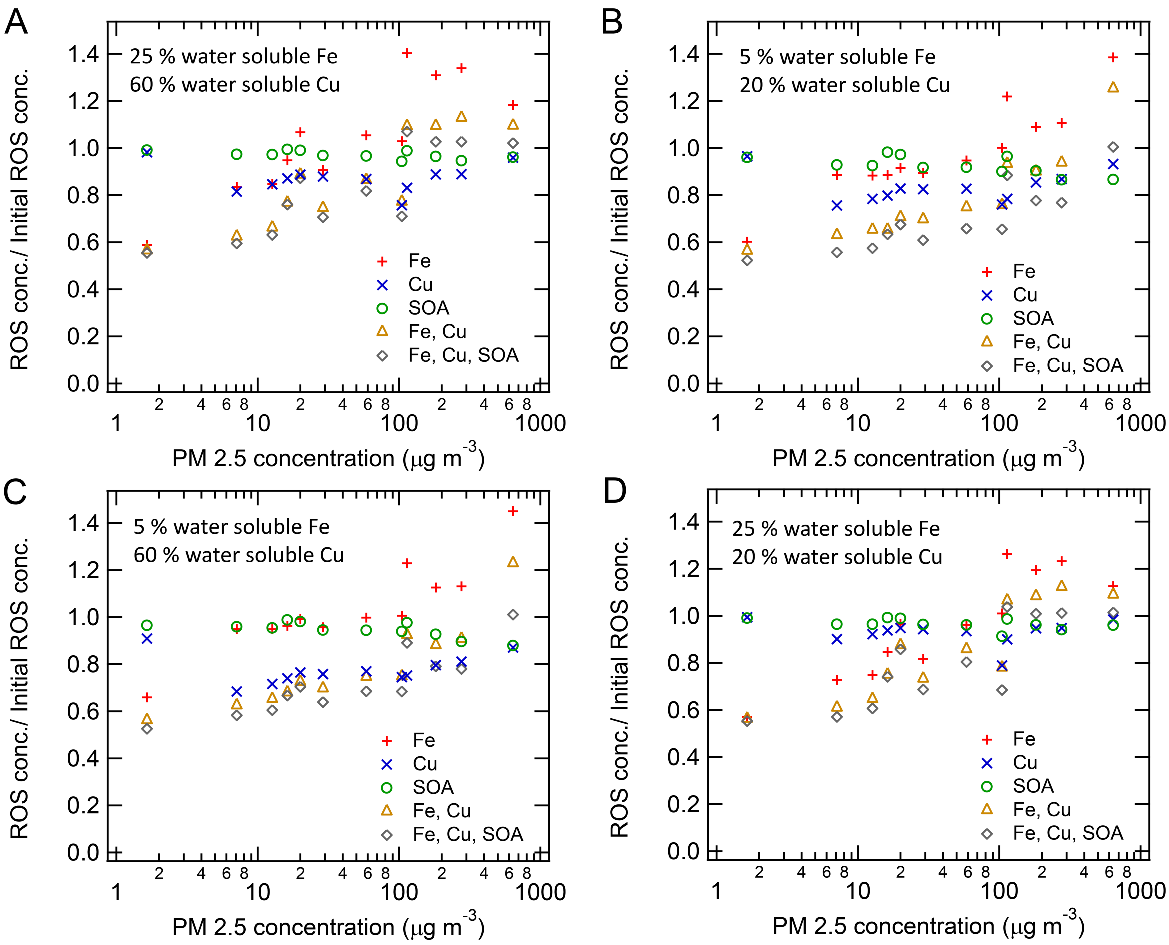

Supplementary Figure 3: Sensitivity studies of the effect of iron and copper solubility upon the importance of removal of 50 % of different redox-active components of PM2.5 and the results shown in Figure 2D. The soluble fractions of iron and copper were assumed to be (A) 25 % for Fe and 60 % for Cu, (B) 5 % for Fe and 20 % for Cu, (C) 5 % for Fe and 60 % for Cu and (D) 25 % for Fe and 20 % for Cu.

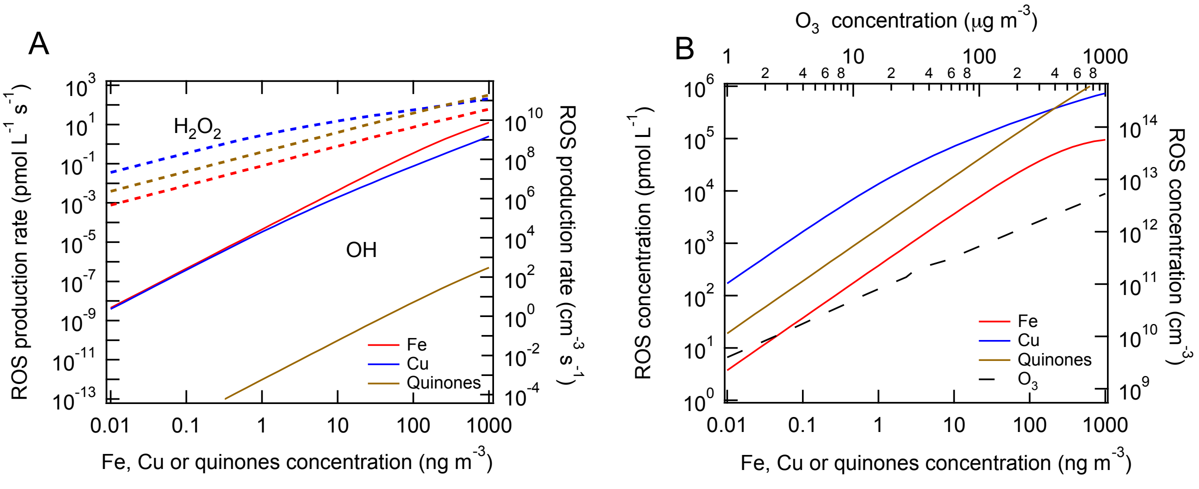

Supplementary Figure 4: (A) The OH and H_2O_2 production rates as a function of different concentrations of Fe, Cu or quinones and (B) ROS (H_2O_2 + OH + O_2^- + HO_2 + O_3) concentrations in the bronchi after 2 hours of exposure to Fe, Cu, quinones or O_3. Note that for all simulations each component was varied with the other concentrations set to zero.

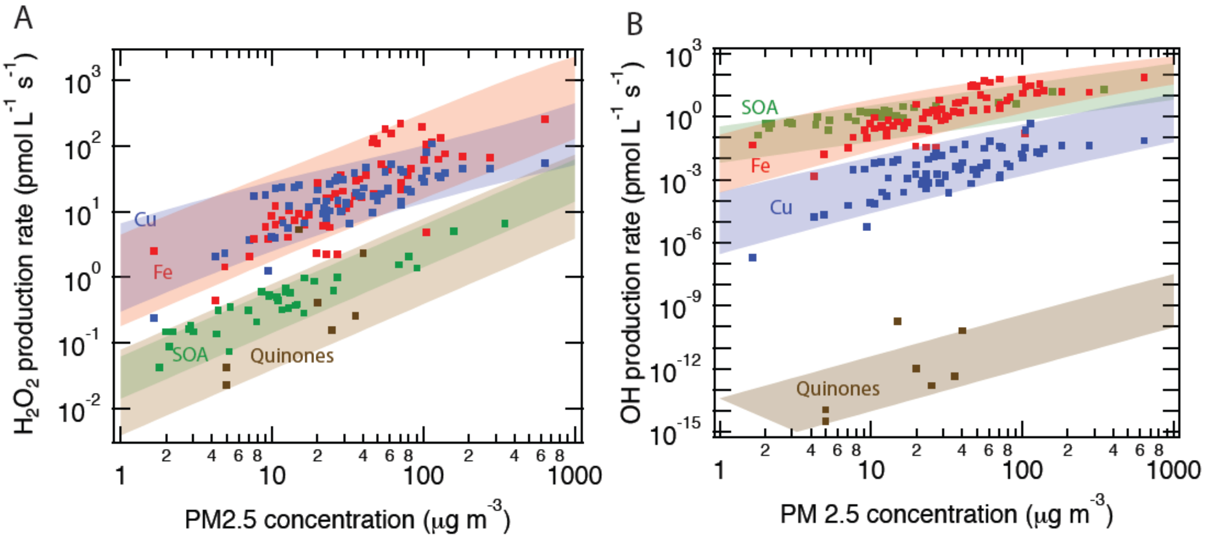

Supplementary Figure 5: Chemical exposure-response relations between air pollutants and ROS in the human respiratory tract. (A) PM2.5 – H_2O_2 production rate corridors (i.e. the range of production rates induced by SOA, Fe, Cu, and quinones contained in PM2.5, respectively). (B) PM2.5 - OH production rate corridors. Different cities are shown by markers (see SI text and Supplementary Tables 4 – 6 for more details).

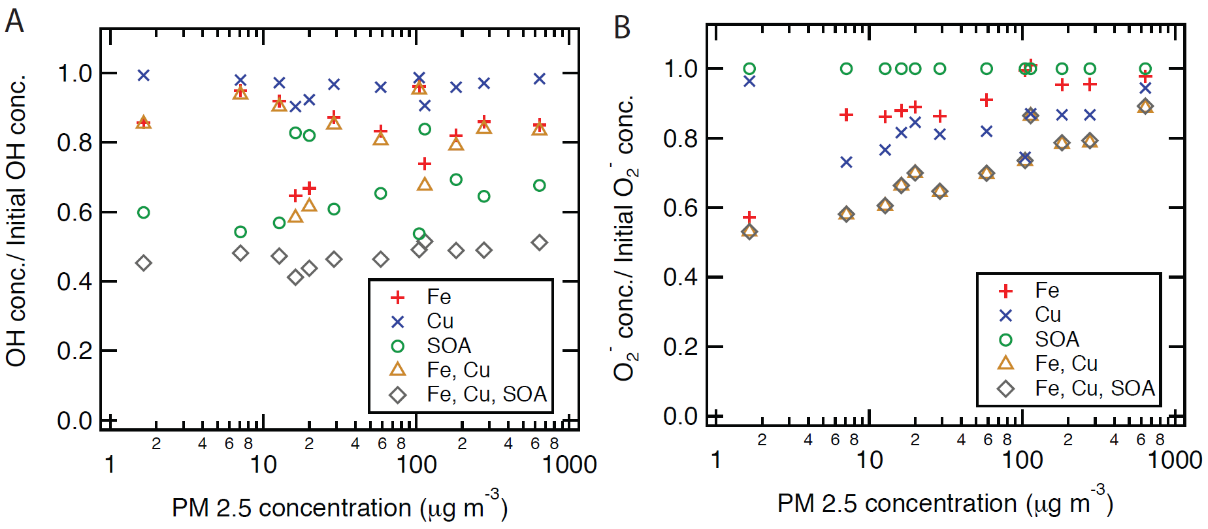

Supplementary Figure 6: Fractional change of (A) OH and (B) O_2^- concentrations in the ELF upon removal of 50% of redox-active components from PM2.5 calculated for selected geographic locations with different PM2.5 concentration levels and composition (Supplementary Table 7).

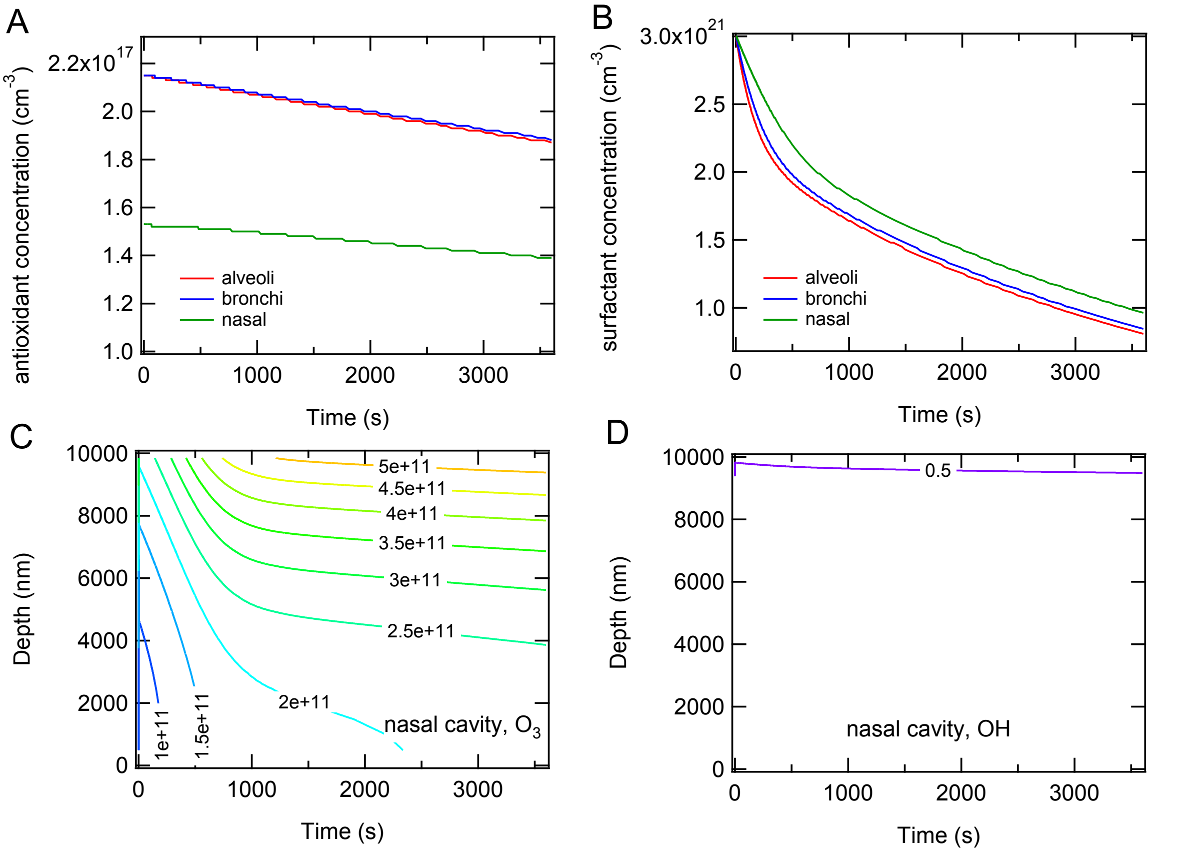

Supplementary Figure 7: Temporal evolution of antioxidants, surfactants and oxidants when exposed to 100 ppb O_3 and 5×10^6 cm^{-3} OH for 1 h. (A) Total antioxidant concentrations in the nasal cavity (green), bronchi (blue) and alveoli (red). (B) Concentrations of surfactants in the nasal cavity, bronchi and alveoli. Bulk concentration profiles of ozone (C) and OH (D) within the ELF in the nasal cavity.

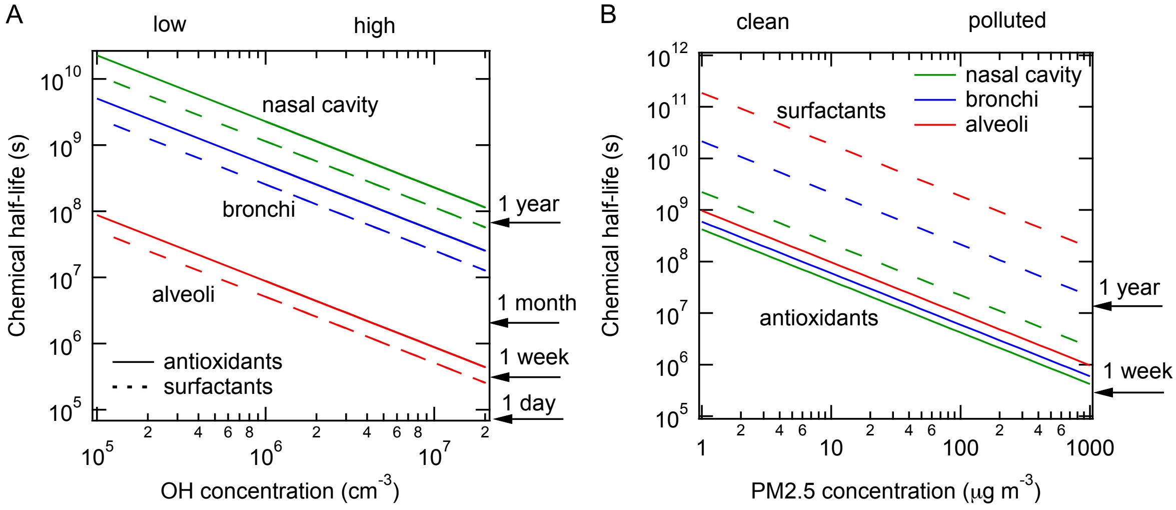

Supplementary Figure 8: Chemical half-life of antioxidants and surfactants in the epithelial lining fluid of the nasal cavity (green), bronchi (blue), and alveoli (red) as a function of (A) ambient concentrations of OH and (B) PM2.5. Deposition of PM2.5 within ELF can lead to formation of OH radicals, which can react with antioxidants and surfactants.

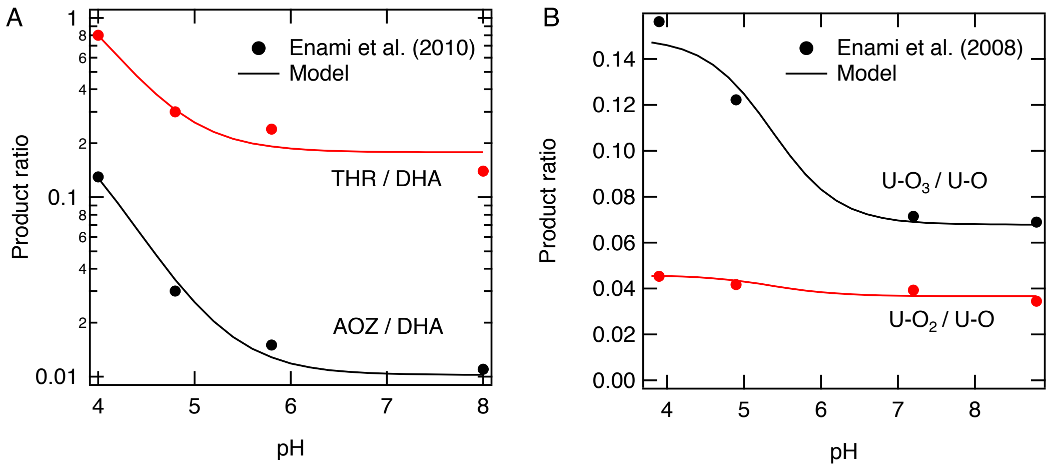

Supplementary Figure 9: (A) Measured[70] and modeled ratio of products of the reactions between ozone and ascorbic acid as a function of pH: threonate (THR) / dehydroascorbic acid (DHA) (red) and ascorbate ozonide (AOZ) / DHA (black). (B) Measured[71] and modeled ratio of products of the reactions between ozone and uric acid as a function of pH: uric acid ozonide (U- O_3) / epoxide (U-O) (black) and uric acid peroxide (U-O_2) / U-O (red).

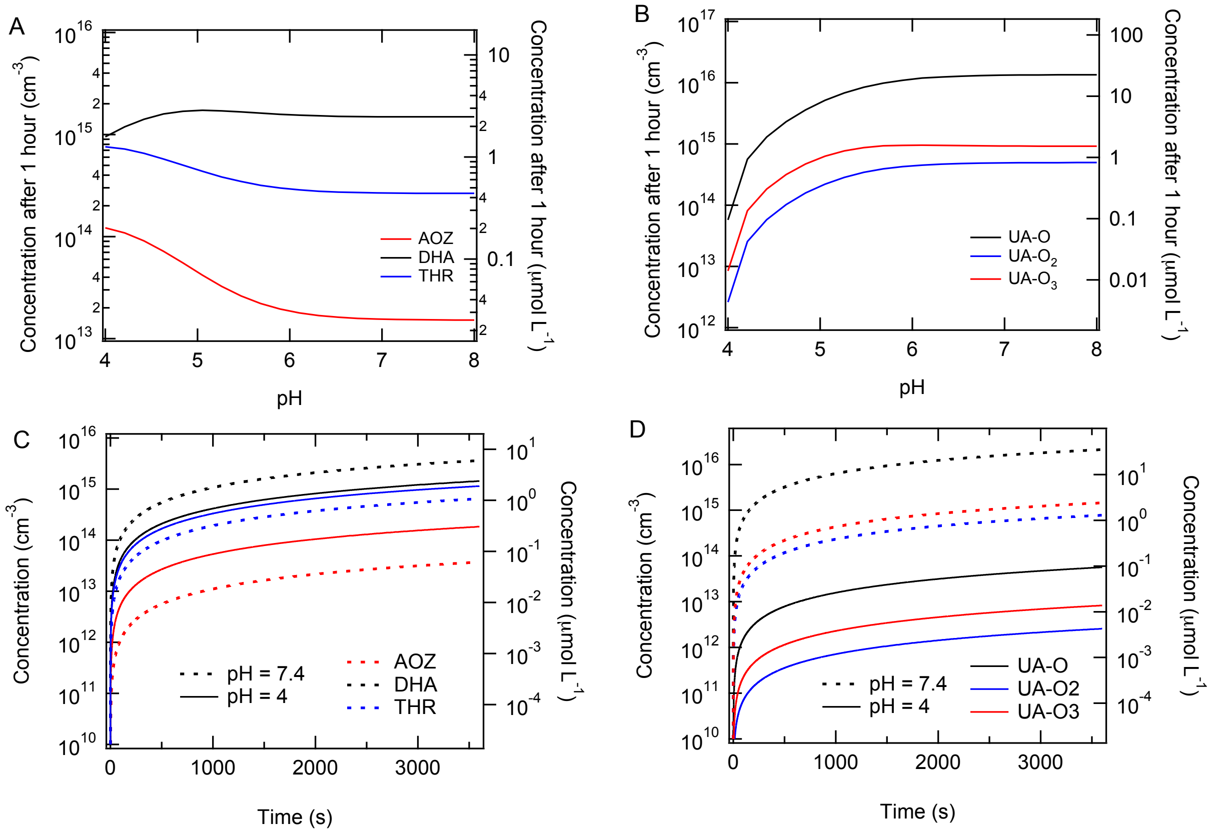

Supplementary Figure 10: pH effect on oxidation products of ozonolysis of ascorbate (A, C) and uric acid (B, D) within ELF in bronchi after one-hour exposure of 100 ppb O_3. (A) Concentrations of ascorbate ozonide (AOZ), dehydroascorbic acid (DHA), and threonate (THR) as a function of pH. (B) Concentrations of uric acid epoxide (UA-O), peroxide (UA-O_2), and ozonide (UA-O_3) as a function of pH. Temporal evolution of ozonolysis products of (C) ascorbate and (D) uric acid at an ELF pH of 7.4 (dashed lines) and pH 4 (solid lines).

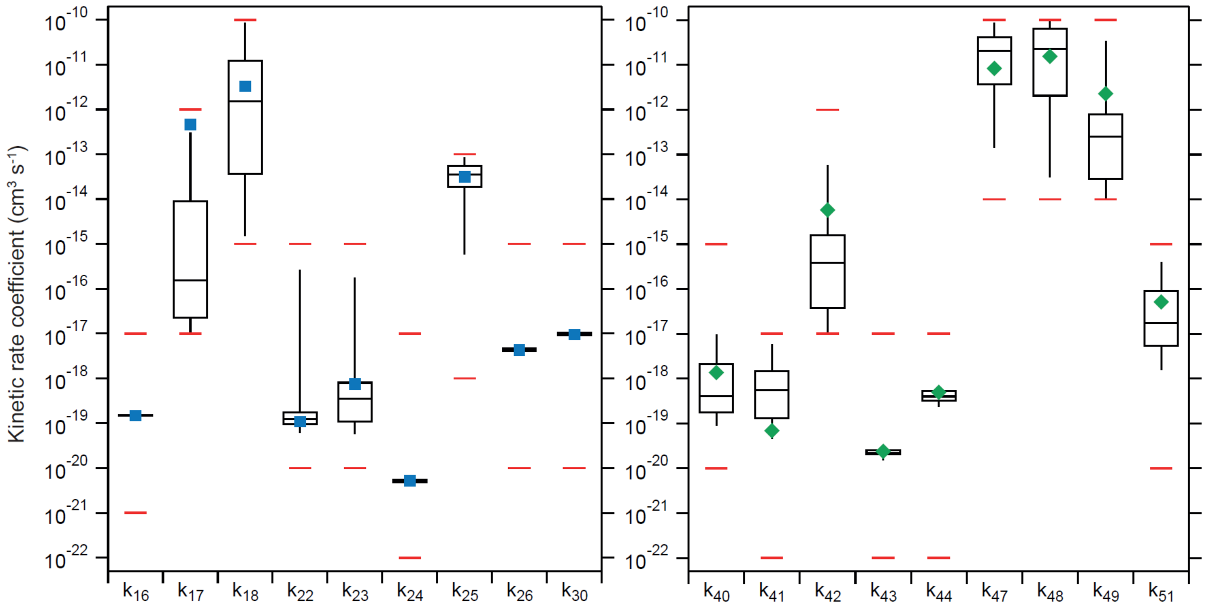

Supplementary Figure 11: Ranges for kinetic parameters obtained with global optimization to experimental data[3,4]. Multiple optimizations for reactions involving iron (left, N=210) and copper (right, N=38) yield estimations of fit parameter uncertainty. Black boxes denote the .25 to .75 percentile of all obtained fit parameters and black whiskers denote the .05 and .95 percentile. The median values are given as black horizontal line. Red markers indicate the fitting boundaries; blue squares and green diamonds represent the best fit to the full kinetic data set.

Supplementary Table 1: A summary of the chemical reactions and rate coefficients included in the KM-SUB-ELF model.

Reaction number	Reaction	Rate coefficient/ $cm^3\ s^{-1}$	Reference
**O_3 reactions**			
1	$Asc + O_3 \rightarrow DHA + AOZ + THR$	$k_1 = 9.1 \times 10^{-17}$	65
2	$UA + O_3 \rightarrow UA\text{-}O + UA\text{-}O_2 + UA\text{-}O_3$	$k_2 = 9.6 \times 10^{-17}$	65
3	$1.25GSH + 0.5O_3 \rightarrow 0.5(GSO_3^{-} + GSO_3^{2-})$	$k_3 = 9.6 \times 10^{-20}$ +	65
4	$\alpha\text{-Toc} + O_3 \rightarrow \alpha\text{-TO} + \alpha\text{-TO}_2 + \alpha\text{-TO}_3 + \alpha\text{-TO}_4 + \alpha\text{-TO}_5$	$k_4 = 1.2 \times 10^{-18}$ ++	65
5	$SP\text{-}B_{1\text{-}25} + O_3 \rightarrow$ Products	$k_5 = 1.0 \times 10^{-14}$	67,80
6	$POG + O_3 \rightarrow$ Products	$k_6 = 4.5 \times 10^{-16}$	21
**OH reactions**			
7	$Asc + OH \rightarrow$ Products	$k_7 = 1.8 \times 10^{-11}$	81
8	$UA + OH \rightarrow$ Products	$k_8 = 1.2 \times 10^{-11}$	82
9	$GSH + OH \rightarrow$ Products	$k_9 = 1.7 \times 10^{-11}$	83,84
10	$\alpha\text{-Toc} + OH \rightarrow$ Products	$k_{10} = 4.5 \times 10^{-13}$	85
11	$SP\text{-}B_{1\text{-}25} + OH \rightarrow$ Products	$k_{11} = 1.7 \times 10^{-11}$	86-88
12	$POG + OH \rightarrow$ Products	$k_{12} = 1.7 \times 10^{-11}$	Assumed to be the same as R11
**Reactions involving semiquinones**			
13	$PQN + Asc \rightarrow PQN^{\cdot} + Asc^{\cdot}$	$k_{13} = 1.2 \times 10^{-20}$	3
14	$PQN^{\cdot} + O_2 \rightarrow PQN + {}^{\cdot}O_2^{-}$	$k_{14} = 4.6 \times 10^{-13}$	See text & Supplementary Fig. 11
15	$PQN^{\cdot} + {}^{\cdot}O_2^{-} + 2H^{+} \rightarrow PQN + H_2O_2$	$k_{15} = 3.3 \times 10^{-12}$	See text & Supplementary Fig. 11
16	$1,2\text{-}NQN + Asc \rightarrow 1,2\text{-}NQN^{\cdot}$ +	$k_{16} = 1.5 \times 10^{-19}$	3

	Asc$^{\cdot}$		
17	1,2-NQN$^{\cdot}$ + O_2 → 1,2-NQN + $^{\cdot}O_2^-$	$k_{17} = 4.6 \times 10^{-13}$	See text & Supplementary Fig. 11
18	1,2-NQN$^{\cdot}$ + $^{\cdot}O_2^-$ + $2H^+$ → 1,2-NQN + H_2O_2	$k_{18} = 3.3 \times 10^{-12}$	See text & Supplementary Fig. 11
19	1,4-NQN + Asc → 1,4-NQN$^{\cdot}$ + Asc$^{\cdot}$	$k_{19} = 6.3 \times 10^{-21}$	3
20	1,4-NQN$^{\cdot}$ + O_2 → 1,4-NQN + $^{\cdot}O_2^-$	$k_{20} = 4.6 \times 10^{-13}$	See text & Supplementary Fig. 11
21	1,4-NQN$^{\cdot}$ + $^{\cdot}O_2^-$ + $2H^+$ → 1,4-NQN + H_2O_2	$k_{21} = 3.3 \times 10^{-12}$	See text & Supplementary Fig. 11

Reactions involving iron

22	Asc + Fe^{3+} → Asc$^{\cdot}$ + Fe^{2+}	$k_{22} = 1.1 \times 10^{-19}$	See text & Supplementary Fig. 11
23	Asc + Fe^{IV} → Asc$^{\cdot}$ + Fe^{3+}	$k_{23} = 7.6 \times 10^{-19}$	See text & Supplementary Fig. 11
24	Fe^{2+} + O_2 → $^{\cdot}O_2^-$ + Fe^{3+}	$k_{24} = 5.2 \times 10^{-21}$	See text & Supplementary Fig. 11
25	Fe^{2+} + $^{\cdot}O_2^-$ + $2H^+$ → Fe^{3+} + H_2O_2	$k_{25} = 3.1 \times 10^{-14}$	See text & Supplementary Fig. 11
26	Fe^{2+} + H_2O_2 → Fe^{3+} + OH^- + OH	$k_{26} = 4.3 \times 10^{-18}$	See text & Supplementary Fig. 11
27	Fe^{2+} + OH → Fe^{3+} + OH^-	$k_{27} = 5.3 \times 10^{-13}$	89
28	Fe^{3+} + H_2O_2 → Fe^{2+} + HO_2 + H^+	$k_{28} = 3.3 \times 10^{-24}$	90
29	Fe^{3+} + HO_2 → Fe^{2+} + O_2 + H^+	$k_{29} = 3.3 \times 10^{-18}$ +++	91
30	Fe^{2+} + H_2O_2 → $Fe^{IV}O^{2+}$ + H_2O	$k_{30} = 9.5 \times 10^{-18}$	See text & Supplementary Fig. 11
31	Fe^{IV} + Fe^{2+} → $2Fe^{3+}$	$k_{31} = 6.6 \times 10^{-18}$	92

Reactive oxygen radical reactions

32	H_2O_2 + OH → H_2O + HO_2	$k_{32} = 5.5 \times 10^{-14}$	93
33	OH + OH → H_2O_2	$k_{33} = 8.6 \times 10^{-12}$	94
34	OH + HO_2 → H_2O + O_2	$k_{34} = 1.2 \times 10^{-11}$	94
35	HO_2 + HO_2 → H_2O_2 + O_2	$k_{35} = 1.4 \times 10^{-15}$	91

36	$H_2O_2 + HO_2 \rightarrow H_2O + O_2 + OH$	$k_{36} = 5.0 \times 10^{-21}$	95
37	$HO_2 + O_2^- \rightarrow H_2O_2 + OH^- + O_2$	$k_{37} = 1.7 \times 10^{-13}$	91
38	$^{\cdot}O_2^- + OH \rightarrow O_2 + OH^-$	$k_{38} = 1.3 \times 10^{-11}$	86
39	$HO_2 \rightleftharpoons H^+ + {}^{\cdot}O_2^-$	$K_{eq} = 2.1 \times 10^{-5}$	96
		$k_{39,\ bwd} = 1.0 \times 10^{-11}$	97
		$k_{39,\ fwd} = 2.3 \times 10^{5}\ s^{-1}$	

Reactions involving copper

40	$Asc + Cu^{2+} \rightarrow Asc^{\cdot} + Cu^{+}$	$k_{40} = 1.4 \times 10^{-18}$	See text & Supplementary Fig. 11
41	$Cu^{+} + O_2 \rightarrow {}^{\cdot}O_2^- + Cu^{2+}$	$k_{41} = 6.9 \times 10^{-20}$	See text & Supplementary Fig. 11
42	$Cu^{+} + HO_2 + H^{+} \rightarrow Cu^{2+} + H_2O_2$	$k_{42} = 5.8 \times 10^{-15}$	See text & Supplementary Fig. 11
43	$Cu^{+} + H_2O_2 \rightarrow Cu^{2+} + OH^- + OH$	$k_{43} = 2.4 \times 10^{-20}$	See text & Supplementary Fig. 11
44	$Cu^{+} + H_2O_2 \rightarrow Cu^{3+} + 2OH^-$	$k_{44} = 5.0 \times 10^{-19}$	See text & Supplementary Fig. 11
45	$Cu^{+} + Cu^{3+} \rightarrow 2Cu^{2+}$	$k_{45} = 5.8 \times 10^{-12}$	98
46	$Cu^{2+} + H_2O_2 \rightarrow Cu^{+} + HO_2 + H^{+}$	$k_{46} = 3.8 \times 10^{-24}$	See text & Supplementary Fig. 11
47	$Cu^{2+} + {}^{\cdot}O_2^- \rightarrow Cu^{+} + O_2$	$k_{47} = 8.3 \times 10^{-12}$	See text & Supplementary Fig. 11
48	$Cu^{2+} + HO_2 \rightarrow Cu^{+} + O_2 + H^{+}$	$k_{48} = 1.6 \times 10^{-11}$	See text & Supplementary Fig. 11
49	$Cu^{+} + HO_2 \rightarrow H_2O_2 + Cu^{2+} + OH^-$	$k_{49} = 2.3 \times 10^{-12}$	See text & Supplementary Fig. 11

Other reactions

50	$2\ Asc^{\cdot} + H^{+} \rightarrow Asc + DHA$	$k_{50} = 5.0 \times 10^{-16}$	99
51	$Asc + O_2^- \rightarrow Asc^{\cdot} + H_2O_2$	$k_{51} = 5.1 \times 10^{-17}$	See text & Supplementary Fig. 11
52	$OH + Benzoate \rightarrow p\text{-}HBA$	$k_{52} = 5.0 \times 10^{-12}$	100
53	$^{\cdot}O_2^- + O_2^- + 2H^{+} \xrightarrow{SOD} H_2O_2 + O_2$	$k_{53} = 2.7 \times 10^{-12}$	101

Asc, UA, GSH and α-Toc represent both the protonated and deprotonated species which will be present in different quantities at different pH.

[+] Units are $cm^{2.25}\,s^{-1}$.

[++] This is a maximum value based upon $k_1/\,k_4 > 75$.

[+++] This is a maximum value.

Supplementary Table 2: Input parameters of the KM-SUB-ELF model.

Parameter	Description	Value
$\alpha_{s,0,OH}$, $\alpha_{s,0,O3}$, $\alpha_{s,0,O2}$	Surface mass accommodation of OH O_3 and O_2 at time zero	1
H_{O3}	Henry's law constant of O_3	1×10^{-5} mol cm^{-3} atm^{-1} [a]
H_{OH}	Henry's law constant of OH	0.03 mol cm^{-3} atm^{-1} [a]
H_{O2}	Henry's law constant of O_2	1.3×10^{-6} mol cm^{-3} atm^{-1} [a]
$D_{b,OH}$, $D_{b,O3}$, $D_{b,O2}$	Bulk diffusion coefficient of OH, O_3 and O_2	1×10^{-5} cm^2 s^{-1}
$D_{b,AO}$, $D_{b,Products}$	Bulk diffusion coefficient of antioxidants and products	1×10^{-7} cm^2 s^{-1}
$\tau_{d,OH}$, $\tau_{d,O3}$, $\tau_{d,O2}$	Desorption lifetime of OH, O_3 and O_2	10^{-9} s
σ_{O3}	Effective molecular cross-section of O_3	4.8×10^{-15} cm
σ_{OH}	Effective molecular cross-section of OH	2.4×10^{-15} cm
σ_{O2}	Effective molecular cross-section of O_2	3.7×10^{-15} cm
ω_{O3}	Mean thermal velocity of O_3	3.6×10^4 cm s^{-1}
ω_{OH}	Mean thermal velocity of OH	6.1×10^4 cm s^{-1}
ω_{O2}	Mean thermal velocity of O_2	4.4×10^4 cm s^{-1}
$D_{g,O3}$, $D_{g,OH}$	Gas phase diffusion coefficient of O3 and OH	0.23 cm^2 s^{-1}

[a] Values are from reference [102].

Supplementary Table 3: Epithelial lining fluid characteristics: literature values of the ELF thickness, antioxidant concentrations, surface area and diameter of respiratory tract in the nasal cavity, bronchi and alveoli.

	Nasal cavity	Bronchi	Alveoli
ELF thickness (µm)	10	0.5	0.05
Antioxidant conc.*			
Ascorbate (µM)	28	40	40
Uric acid (µM)	225	207	207
Glutathione (µM)	0.5	109	109
α-Tocopherol (µM)	0.7	0.7	0.7
Surface area (cm^2)**	180	4500	885000
Diameter of the respiratory tract (cm)***	4.5	1	0.02

* Mudway and Kelly[103].

** Cross *et al.*[18].

*** Ochs *et al.*[104], Franciscus and Long[105].

Supplementary Table 4: Properties and effects of fine particulate matter with particle diameters less than 2.5 µm (PM2.5) at various locations: measured mass concentrations of PM2.5, Fe, and Cu in ambient air and calculated production rates and concentrations of ROS, after two hours of exposure, in the epithelial lining fluid (ELF).

Location	Ambient mass concentrations			Ambient mass fractions		References	ELF production rates						ELF concentrations			
	PM2.5 (µg m^{-3})	Fe (ng m^{-3})*	Cu (ng m^{-3})*	Fe (%)	Cu (%)		OH from Fe (pmol L^{-1} s^{-1})	OH from Cu (pmol L^{-1} s^{-1})	H_2O_2 from Fe (pmol L^{-1} s^{-1})	H_2O_2 from Cu (pmol L^{-1} s^{-1})	ROS from Fe (pmol L^{-1} s^{-1})	ROS from Cu (pmol L^{-1} s^{-1})	OH (nmol L^{-1})**	HO_2 (nmol L^{-1}) **	O_2^- (nmol L^{-1}) **	Total ROS (≈ H_2O_2) (nmol L^{-1}) **
Amazon (wet season)	1.65	33	0.07	2.0	0.004	106	4.42×10^{-2}	1.86×10^{-7}	2.50	2.43×10^{-1}	4.65×10^{2}	47.35	1.53×10^{-10}	2.17×10^{-6}	2.05×10^{-3}	12.80
Mace Head	~4.2	~5.68	~0.71	0.13	0.02	38	1.41×10^{-3}	1.65×10^{-5}	0.44	2.07	85.02	4.03×10^{2}	3.15×10^{-10}	2.15×10^{-6}	2.02×10^{-3}	13.46
Amazon (dry season)	4.87	19	0.80	0.39	0.02	106	1.52×10^{-2}	2.14×10^{-5}	1.45	2.33	2.76×10^{2}	4.53×10^{2}	3.83×10^{-10}	2.82×10^{-6}	2.65×10^{-3}	17.46
Edinburgh	7.1	27.6	1.39	0.39	0.02	37	3.14×10^{-2}	6.00×10^{-5}	2.10	3.67	3.94×10^{2}	7.13×10^{2}	5.74×10^{-10}	4.17×10^{-6}	3.93×10^{-3}	25.77
West Midlands (UK, rural, median)	7.6	51.3	13.5	0.68	0.18	8	1.02×10^{-1}	3.04×10^{-3}	3.84	17.57	6.98×10^{2}	3.34×10^{3}	8.38×10^{-10}	1.57×10^{-5}	1.48×10^{-2}	91.97
West Midlands (UK, urban, median)	9.0	80.2	13.9	0.89	0.15	8	2.32×10^{-1}	3.19×10^{-3}	5.88	17.89	1.03×10^{3}	3.40×10^{3}	1.11×10^{-9}	1.65×10^{-5}	1.56×10^{-2}	96.15
Skukuza (South Africa)	9.4	51	0.41	0.54	0.04	107	1.01×10^{-1}	5.75×10^{-6}	3.82	1.27	6.94×10^{2}	2.48×10^{2}	7.83×10^{-10}	3.87×10^{-6}	3.64×10^{-3}	24.84
Amazon (Serro do Navio)	9.87	120	1.65	1.21	0.02	108	4.83×10^{-1}	7.88×10^{-5}	8.64	4.13	1.45×10^{3}	8.01×10^{2}	1.11×10^{-9}	8.27×10^{-6}	7.80×10^{-3}	48.69
Amazon (Cuiabá)	10.5	175	1.55	1.67	0.01	108	2.70×10^{-1}	5.96×10^{-3}	6.35	22.51	1.11×10^{3}	4.24×10^{3}	1.31×10^{-9}	1.86×10^{-5}	1.76×10^{-2}	1.09×10^{2}
West Midlands (UK, rural, average)	10.5	87.1	20	0.83	0.19	8	9.21×10^{-1}	7.09×10^{-5}	12.24	3.94	1.94×10^{3}	7.66×10^{2}	1.41×10^{-9}	9.89×10^{-6}	9.32×10^{-3}	56.80

West Midlands (UK, urban, average)	11.6	102	21.9	0.88	0.19	8	3.58×10^{-1}	6.92×10^{-3}	7.38	23.76	1.26×10^{3}	4.46×10^{3}	1.5×10^{-9}	1.96×10^{-5}	1.85×10^{-2}	1.15×10^{2}
Helsinki	11.8	96	3.1	0.81	0.03	109	3.24×10^{-1}	2.54×10^{-4}	7.00	6.72	1.20×10^{3}	1.30×10^{3}	1.2×10^{-9}	8.67×10^{-6}	8.18×10^{-3}	51.84
Tampa (Florida)	12.7	79	2.4	0.62	0.02	110	2.26×10^{-1}	1.63×10^{-4}	5.79	5.60	1.02×10^{3}	1.08×10^{3}	1.09×10^{-9}	6.7×10^{-6}	6.31×10^{-3}	41.63
Toronto	12.7	55	2.5	0.43	0.02	111	1.16×10^{-1}	1.73×10^{-4}	4.11	5.75	7.43×10^{2}	1.11×10^{3}	1.65×10^{-9}	1.34×10^{-5}	1.26×10^{-2}	77.37
South Phoenix	12.95	147	7.6	1.14	0.06	112	6.85×10^{-1}	1.20×10^{-3}	10.42	12.38	1.70×10^{3}	2.38×10^{3}	1.18×10^{-9}	7.44×10^{-6}	7.01×10^{-3}	45.52
Tehran (inside a school dormitory)	14	102.13	22.17	0.73	0.16	113	3.62×10^{-1}	7.01×10^{-3}	7.42	23.87	1.27×10^{3}	4.48×10^{3}	1.69×10^{-9}	1.97×10^{-5}	1.86×10^{-2}	1.16×10^{2}
Tehran (inside a retirement home)	15	130.78	25.1	0.87	0.17	113	5.60×10^{-1}	8.59×10^{-3}	9.35	25.69	1.55×10^{3}	4.80×10^{3}	1.99×10^{-9}	2.13×10^{-5}	2.01×10^{-2}	1.25×10^{2}
South-Eastern Italy (background sites)	16.4	86.8	3.1	0.53	0.02	11	2.70×10^{-1}	2.54×10^{-4}	6.35	6.72	1.11×10^{3}	1.30×10^{3}	1.5×10^{-9}	8.42×10^{-6}	7.94×10^{-3}	52.05
Fukue island (Japan)	16.5	102	Unknown	0.62	Unknown	114	3.58×10^{-1}	N/A	7.38	N/A	1.26×10^{3}	N/A	N/A	N/A	N/A	N/A
Patras	17.4	124	7.28	0.71	0.04	36	5.09×10^{-1}	1.12×10^{-3}	8.89	12.05	1.48×10^{3}	2.32×10^{3}	1.86×10^{-9}	1.27×10^{-5}	1.20×10^{-2}	75.44
Yeongwol (Korea)	19.7	31.2	9.8	0.16	0.05	115	3.97×10^{-2}	1.84×10^{-3}	2.37	14.56	4.42×10^{2}	2.79×10^{3}	1.6×10^{-9}	1.22×10^{-5}	1.15×10^{-2}	75.66
Budapest	20	430	18.4	2.15	0.09	116	3.85	5.22×10^{-3}	27.76	21.44	3.56×10^{3}	4.05×10^{3}	4.23×10^{-9}	2.27×10^{-5}	2.14×10^{-2}	1.19×10^{2}
South-Eastern Italy (industrial sites)	21.7	85	5.1	0.39	0.02	11	2.59×10^{-1}	6.07×10^{-4}	6.22	9.52	1.09×10^{3}	1.84×10^{3}	1.94×10^{-9}	1.02×10^{-5}	9.58×10^{-3}	63.47
Zabrze (Upper Silesia, Poland)	22	160.8	6.5	0.73	0.03	117	7.97×10^{-1}	9.22×10^{-4}	11.31	11.20	1.82×10^{3}	2.15×10^{3}	2.38×10^{-9}	1.31×10^{-5}	1.23×10^{-2}	77.92
Chuncheon (Korea)	23	29.6	9.9	0.13	0.04	115	3.58×10^{-2}	1.87×10^{-3}	2.25	14.66	4.20×10^{2}	2.80×10^{3}	1.84×10^{-9}	1.23×10^{-5}	1.16×10^{-2}	77.20
Detroit	23	234	6	1.02	0.03	118	1.49	8.02×10^{-4}	15.99	10.61	2.40×10^{3}	2.04×10^{3}	2.86×10^{-9}	1.44×10^{-5}	1.36×10^{-2}	83.04
Megalopolis	23	87	4.02	0.38	0.02	36	2.70×10^{-1}	4.03×10^{-4}	6.35	8.09	1.11×10^{3}	1.56×10^{3}	2.03×10^{-9}	9.33×10^{-6}	8.79×10^{-3}	59.20

Tehran (outside a retirement home)	24	238.81	25.99	1	0.11	113	1.54	9.08×10^{-3}	16.30	26.21	2.43×10^{3}	4.89×10^{3}	3.43×10^{-9}	2.3×10^{-5}	2.17×10^{-2}	1.32×10^{2}
South-Eastern Italy (urban sites)	24.1	78.8	5.7	0.33	0.02	11	2.26×10^{-1}	7.35×10^{-4}	5.79	10.25	1.02×10^{3}	1.98×10^{3}	2.11×10^{-9}	1.05×10^{-5}	9.88×10^{-3}	66.21
Tehran (outside a school dormitory)	26	280.28	32.42	1.08	0.12	113	2.00	1.29×10^{-2}	18.86	29.69	2.72×10^{3}	5.48×10^{3}	3.99×10^{-9}	2.53×10^{-5}	2.39×10^{-2}	1.44×10^{2}
Anaheim	26.8	29.6	39.6	0.11	0.15	119	3.58×10^{-2}	1.77×10^{-2}	2.25	33.25	4.20×10^{2}	6.08×10^{3}	2.22×10^{-9}	2.5×10^{-5}	2.35×10^{-2}	1.57×10^{2}
Milan (summer)	27.2	186	10	0.68	0.04	120	1.02	1.90×10^{-3}	12.96	14.76	2.03×10^{3}	2.82×10^{3}	3.02×10^{-9}	1.57×10^{-5}	1.48×10^{-2}	93.07
Jeddah City (Saudi Arabia)	28.4	590	5.6	2.08	0.02	121	6.09	7.16×10^{-4}	36.91	10.15	4.27×10^{3}	1.96×10^{3}	5.43×10^{-9}	2.04×10^{-5}	1.92×10^{-2}	1.01×10^{2}
Hong Kong	29	140	5.7	0.48	0.02	122	6.31×10^{-1}	7.35×10^{-4}	9.97	10.25	1.64×10^{3}	1.98×10^{3}	2.79×10^{-9}	1.21×10^{-5}	1.14×10^{-2}	75.22
Rio de Janiero	29.2	307	35	1.05	0.12	123	2.30	1.46×10^{-2}	20.48	31.03	2.89×10^{3}	5.71×10^{3}	4.47×10^{-9}	2.63×10^{-5}	2.48×10^{-2}	1.49×10^{2}
Katowice (Upper Silesia,Poland)	31	157	8.2	0.51	0.03	117	7.66×10^{-1}	1.36×10^{-3}	11.07	13.00	1.79×10^{3}	2.49×10^{3}	3.09×10^{-9}	1.41×10^{-5}	1.33×10^{-2}	86.42
Porto Marghera (Italy)	31	200	9.3	0.65	0.03	9	1.16	1.69×10^{-3}	13.87	14.10	2.15×10^{3}	2.70×10^{3}	3.37×10^{-9}	1.56×10^{-5}	1.47×10^{-2}	93.19
Erzgebirge (Germany)	32.5	188	3	0.58	0.01	124	1.04	2.41×10^{-4}	13.08	6.58	2.05×10^{3}	1.27×10^{3}	3.22×10^{-9}	1.14×10^{-5}	1.08×10^{-2}	71.02
Barcelona	35	260	52	0.74	0.15	125	1.77	2.72×10^{-2}	17.60	38.73	2.58×10^{3}	6.96×10^{3}	4.79×10^{-9}	3×10^{-5}	2.83×10^{-2}	1.77×10^{2}
Santa Catarina (Mexico)	36.15	466	16	1.29	0.04	126	4.34	4.13×10^{-3}	29.85	19.69	3.74×10^{3}	3.73×10^{3}	5.66×10^{-9}	2.24×10^{-5}	2.12×10^{-2}	1.19×10^{2}
Escobedo (Mexico)	37.78	493	13	1.3	0.03	126	4.71	2.94×10^{-3}	31.41	17.36	3.86×10^{3}	3.30×10^{3}	5.86×10^{-9}	2.18×10^{-5}	2.05×10^{-2}	1.14×10^{2}
Arnhem	38.95	241	13.5	0.62	0.03	57	1.57	3.13×10^{-3}	16.45	17.77	2.45×10^{3}	3.38×10^{3}	4.35×10^{-9}	1.84×10^{-5}	1.74×10^{-2}	1.09×10^{2}
Guangzhou (rainy season)	40.18	Unknown	21.28	Unknown	0.05	10	N/A	6.56×10^{-3}	N/A	23.31	N/A	4.38×10^{3}	N/A	N/A	N/A	N/A

Mira Loma (Southern California, average)	41.8	581	75	1.39	0.18	127	5.96	4.82×10^{-2}	36.42	47.33	4.23×10^{3}	8.25×10^{3}	8.16×10^{-9}	3.57×10^{-5}	3.37×10^{-2}	1.91×10^{2}
Taif (Saudi Arabia, residential area)	46	2000	5.3	4.35	0.01	128	28.45	6.48×10^{-4}	1.12×10^{2}	9.76	7.21×10^{3}	1.88×10^{3}	1.47×10^{-8}	3.21×10^{-5}	3.02×10^{-2}	1.08×10^{2}
Taif (Saudi Arabia, industrial site)	47	2300	13	4.89	0.03	128	33.22	2.94×10^{-3}	1.28×10^{2}	17.36	7.53×10^{3}	3.30×10^{3}	1.66×10^{-8}	3.43×10^{-5}	3.24×10^{-2}	1.11×10^{2}
Azusa	47.1	281.9	13.4	0.6	0.03	119	2.02	3.09×10^{-3}	18.98	17.68	2.73×10^{3}	3.36×10^{3}	5.24×10^{-9}	1.9×10^{-5}	1.80×10^{-2}	1.13×10^{2}
Edison	49.6	1953	10	3.94	0.02	129	27.65	1.90×10^{-3}	1.10×10^{2}	14.76	7.15×10^{3}	2.82×10^{3}	1.49×10^{-8}	3.25×10^{-5}	3.06×10^{-2}	1.11×10^{2}
Bursa (Turkey)	53	875	15	1.65	0.03	130	10.39	3.72×10^{-3}	52.61	18.94	5.20×10^{3}	3.59×10^{3}	9.51×10^{-9}	2.65×10^{-5}	2.50×10^{-2}	1.21×10^{2}
Karachi (Pakistan, summer)	55.89	3360	56	6.01	0.1	131	50.10	3.06×10^{-2}	1.82×10^{2}	40.36	8.35×10^{3}	7.21×10^{3}	2.4×10^{-8}	4.07×10^{-5}	3.84×10^{-2}	1.20×10^{2}
New Delhi (summer, high traffic location)	58.2	710	20	1.22	0.03	132	7.83	5.96×10^{-3}	43.44	22.51	4.69×10^{3}	4.24×10^{3}	9.08×10^{-9}	2.62×10^{-5}	2.47×10^{-2}	1.30×10^{2}
Milan (winter)	58.6	309	18	0.53	0.03	120	2.33	4.98×10^{-3}	20.60	21.08	2.90×10^{3}	3.98×10^{3}	6.42×10^{-9}	2.13×10^{-5}	2.01×10^{-2}	1.27×10^{2}
Thessaloniki, Greece (cold period)	60.9	2890	93	4.75	0.15	133	42.62	6.71×10^{-2}	1.58×10^{2}	53.16	8.03×10^{3}	9.06×10^{3}	2.31×10^{-8}	4.26×10^{-5}	4.02×10^{-2}	1.34×10^{2}
Thessaloniki, Greece (warm period)	70.6	4094	66	5.8	0.09	133	61.59	3.94×10^{-2}	2.19×10^{2}	44.10	8.74×10^{3}	7.78×10^{3}	2.9×10^{-8}	4.22×10^{-5}	3.98×10^{-2}	1.19×10^{2}
Córdoba City (Argentina)	70.87	325	8	0.46	0.01	134	2.52	1.31×10^{-3}	21.57	12.81	2.99×10^{3}	2.46×10^{3}	7.2×10^{-9}	1.75×10^{-5}	1.65×10^{-2}	1.08×10^{2}
Shanghai	71.61	424.93	9.47	0.59	0.01	135	3.79	1.74×10^{-3}	27.47	14.26	3.54×10^{3}	2.73×10^{3}	7.96×10^{-9}	1.97×10^{-5}	1.86×10^{-2}	1.15×10^{2}
Guangzhou (dry season)	73.58	Unknown	57.89	Unknown	0.08	10	N/A	3.21×10^{-2}	N/A	41.05	N/A	7.32×10^{3}	N/A	N/A	N/A	N/A
Yong'an (China, winter)	79.01	582.1	19.6	0.74	0.02	136	5.98	5.71×10^{-3}	36.49	22.16	4.24×10^{3}	4.17×10^{3}	9.91×10^{-9}	2.5×10^{-5}	2.36×10^{-2}	1.35×10^{2}

Yong'an (China, spring)	83.26	736.1	16.5	0.88	0.02	136	8.29	4.35×10^{-3}	45.15	20.06	4.79×10^{3}	3.80×10^{3}	1.11×10^{-8}	2.57×10^{-5}	2.43×10^{-2}	1.30×10^{2}
Karachi (Pakistan, winter)	98.44	3706	39	3.76	0.04	131	55.56	1.73×10^{-2}	1.99×10^{2}	33.00	8.55×10^{3}	6.04×10^{3}	2.87×10^{-8}	4.01×10^{-5}	3.78×10^{-2}	1.16×10^{2}
Ji'nan (China, urban site)	101	1040	30	1.03	0.03	137	13.02	1.14×10^{-2}	61.65	28.47	5.63×10^{3}	5.28×10^{3}	1.49×10^{-8}	3.1×10^{-5}	2.93×10^{-2}	1.43×10^{2}
Guangzhou (China)	104.58	66	60	0.06	0.06	138	0.16	3.40×10^{-2}	4.87	41.87	8.70×10^{2}	7.44×10^{3}	8.39×10^{-9}	3.08×10^{-5}	2.91×10^{-2}	2.21×10^{2}
Agra (India, urban)	104.9	1900	200	1.81	0.19	139	26.75	2.18×10^{-1}	1.07×10^{2}	80.44	7.08×10^{3}	1.22×10^{4}	2.37×10^{-8}	4.84×10^{-5}	4.57×10^{-2}	1.96×10^{2}
Pune (India)	113.8	2090	339	1.84	0.3	140	29.84	4.85×10^{-1}	1.17×10^{2}	107.60	7.31×10^{3}	1.45×10^{4}	2.74×10^{-8}	5.38×10^{-5}	5.09×10^{-2}	2.26×10^{2}
Beijing (summer)	125	1060	44.7	0.85	0.04	141	13.32	2.14×10^{-2}	62.65	35.58	5.67×10^{3}	6.46×10^{3}	1.73×10^{-8}	3.37×10^{-5}	3.18×10^{-2}	1.59×10^{2}
Ji'nan (China, industrial site)	130	2410	40	1.85	0.03	137	35.01	1.81×10^{-2}	1.33×10^{2}	33.50	7.64×10^{3}	6.12×10^{3}	2.49×10^{-8}	3.78×10^{-5}	3.57×10^{-2}	1.28×10^{2}
Beijing (winter)	138	1330	53.2	0.96	0.04	141	17.58	2.82×10^{-2}	76.83	39.24	6.22×10^{3}	7.04×10^{3}	2.02×10^{-8}	3.6×10^{-5}	3.40×10^{-2}	1.58×10^{2}
Beijing	182.2	1180	70	0.65	0.04	142	15.19	4.32×10^{-2}	68.94	45.56	5.93×10^{3}	7.99×10^{3}	2.33×10^{-8}	3.78×10^{-5}	3.56×10^{-2}	1.82×10^{2}
New Delhi (winter, high traffic location)	276.9	1150	70	0.42	0.03	132	14.70	4.32×10^{-2}	67.29	45.56	5.86×10^{3}	7.99×10^{3}	3.07×10^{-8}	3.8×10^{-5}	3.59×10^{-2}	1.99×10^{2}
Peat fire episode (Indonesia)	640	4810	100	0.75	0.02	143	72.80	7.30×10^{-2}	2.55×10^{2}	54.74	9.05×10^{3}	9.27×10^{3}	8.04×10^{-8}	4.62×10^{-5}	4.36×10^{-2}	1.49×10^{2}

* Values within the table represent the total iron and copper concentrations. However, for calculations an iron solubility of 10% and a copper solubility of 40% was assumed.

**ROS concentrations were calculated assuming an average percentage of PM2.5 of 30 % SOA and 0.005% quinones.

Supplementary Table 5: Measured quinone concentrations within PM2.5 in the literature and OH, H_2O_2 and ROS production rates calculated using the KM-SUB-ELF model.

Location	Ambient mass concentrations				Ambient mass fractions	References	ELF production rates		
	PM2.5 (μg m^{-3})	PQN (ng m^{-3})	1,2-NQN (ng m^{-3})	1,4-NQN (ng m^{-3})	(%)		OH (pmol L^{-1} s^{-1})	H_2O_2 (pmol L^{-1} s^{-1})	ROS (pmol L^{-1} s^{-1})
Umea, Sweden	7.8	Not measured	Not measured	0.03	N/A	144	2.32×10^{-17}	1.93×10^{-3}	3.77×10^{-1}
Athens	35.6	0.071	0.157	0.26	1.37×10^{-3}	145	4.22×10^{-13}	2.61×10^{-1}	50.84
Mazar-e Sharif (Afghanistan)	69	Not measured	Not measured	0.027	N/A	144	1.88×10^{-17}	1.74×10^{-3}	3.39×10^{-1}
Kabul (Afghanistan)	86	Not measured	Not measured	0.2	N/A	144	1.03×10^{-15}	1.29×10^{-2}	2.51
Atascadero (southern California)**	~ 5	0.023	0.0127	0.0246	~5.63×10^{-3}	146	3.27×10^{-15}	2.30×10^{-2}	4.48
Birmingham**	~ 15	4.6	3.2	1.7	~0.063	147	1.77×10^{-10}	5.36	1.04×10^{3}
Chiang Mai, Thailand	Not available	Not measured	Not measured	0.051	N/A	148	6.71×10^{-17}	3.29×10^{-3}	6.41×10^{-1}
Lake Elsinore (southern California)**	~ 20	0.311	0.246	0.14	~3.49×10^{-3}	146	1.04×10^{-12}	4.09×10^{-1}	79.75
Norfolk, UK**	~ 5	0.058	0.024	0.012	~1.88×10^{-3}	149	1.12×10^{-14}	4.26×10^{-2}	8.29
Riverside (southern California)**	~ 25	0.57	0.06	0.23	~3.44×10^{-3}	150	1.57×10^{-13}	1.59×10^{-1}	31.04
Salvador, Brazil**	~ 40	< 0.20 - 7.39	< 0.05 - 2.4	< 0.05 - 3.17	~0.016	151	6.22×10^{-14} - 1.31×10^{-10}	0.10 – 4.61	19.52 – 8.94×10^{2}
Tempe	Not available	0.427	Not measured	Identified	N/A	152	9.97×10^{-15}	4.01×10^{-2}	7.82

*PM2.5 concentrations were estimated based on the type of location (i.e. rural, city, megacity) and based upon known values for similar locations within Supplementary Table 4.

Supplementary Table 6: Measured SOA concentrations within submicron aerosols and PM2.5 in the literature and estimated OH H_2O_2 and ROS production rates.

**Location**	**Ambient mass concentrations**		**Ambient mass fractions**	**ELF production rates**		
	**Submicron aerosols or PM2.5 (μg m^{-3})**	**SOA (μg m^{-3})**	**(%)**	**OH (pmol L^{-1} s^{-1})**	**H_2O_2 (pmol L^{-1} s^{-1})**	**ROS (pmol L^{-1} s^{-1})**
Amazon[B]	1.8	0.34	18.9	0.14	0.05	0.18
Hyytiälä (Finland)	2	1.2	60	0.45	0.16	0.61
Storm Peak (CO, USA)	2.1	0.7	33.3	0.27	0.09	0.36
Jungfraujoch (Switzerland)	2.2	1.2	54.5	0.45	0.16	0.61
Duke Forest (NC, USA)	2.8	1.3	46.4	0.50	0.16	0.65
Chebogue Pt. (Canada)	2.9	1.5	51.7	0.56	0.18	0.74
Edinburgh (UK)	3	1.2	40	0.45	0.16	0.61
Mainz (Germany)	4.3	1.1	25.6	0.41	0.14	0.54
Boulder (CO, USA)	4.4	2.5	56.8	0.95	0.32	1.26
Manchester (UK, winter)	5.2	0.6	11.5	0.23	0.07	0.29
Chelmsford (UK)	5.3	1.8	34	0.68	0.36	1.04
Vancouver (Canada)	7	2.5	35.7	0.95	0.32	1.26
Okinawa (Japan)	7.9	1.7	21.5	0.63	0.20	0.83
Off New England Coast	8.5	4.9	57.6	1.85	0.61	2.45
Thompson Farm (NH, USA)	9.5	4.2	44.2	1.58	0.52	2.09
Zurich (Switzerland, winter)	9.6	4.3	44.8	1.62	0.54	2.16
Cheju (Korea)	10.7	4	37.4	1.51	0.50	2.00
Fukue (Japan)	11	3.6	32.7	1.35	0.45	1.80
New York City (USA, winter)	11.6	2.6	22.4	0.97	0.32	1.28
New York City (USA, summer)	12.2	4.8	39.3	1.80	0.61	2.41
Pinnacle Park (NY,	12.3	5.4	43.9	2.03	0.68	2.70

USA)						
Houston (TX, USA)	12.8	2.7	21.1	1.01	0.34	1.35
Tokyo (Japan, summer)	13.2	4.7	35.6	1.76	0.59	2.34
Manchester (UK, summer)	14.3	3	21	1.13	0.38	1.51
Pittsburgh (PA, USA)	14.7	3.1	21.1	1.17	0.38	1.55
Tokyo (Japan, winter)	16.2	2.3	14.2	0.86	0.29	1.15
Taunus (Germany)	16.3	7.9	48.5	2.97	0.99	3.96
Riverside (CA, USA)	19.1	7	36.6	2.63	0.88	3.51
Zurich (Switzerland, summer)	25.5	5.1	20	1.91	0.63	2.54
Mexico City (Mexico)	26.8	8.1	30.2	3.04	1.01	4.05
Guangzhou [A]	69.1	12.5	18.1	4.68	1.55	6.23
Beijing (China)	79.9	16.6	20.8	6.23	2.07	8.30
Shanghai (China)[A]	90.7	11.1	12.2	4.14	1.37	5.51
Beijing (China)[A]	158.5	40.9	25.8	15.35	5.09	20.43
Xi'an [A]	345.1	53.5	15.5	20.07	6.66	26.73

*All concentrations are from Jimenez et al.[153] (and references therein) with the exception of A = Huang et al.[154] and B= Pöschl et al.[155].

Supplementary Table 7: Fractional change of ROS, OH and O_2^- concentrations upon 50% removal of redox-active components from PM2.5: ROS concentration after 50 % removal divided by initial ROS concentration prior to 50 % removal.

Location	Ambient mass concentrations				References		Initial ROS conc.	Conc. / Initial Conc.					
	PM2.5 (μg m^{-3})	Fe (ng m^{-3})	Cu (ng m^{-3})	SOA (μg m^{-3})			(nmol L^{-1}) *	50% Fe	50% Cu	50% SOA	50% quinones	50% Fe and Cu	50% Fe, Cu and SOA
Amazon	1.65	33	0.07	-	106	ROS:	12.9	0.591	0.966	0.979	0.994	0.552	0.530
						OH:	1.53×10^{-10}	0.857	0.993	0.598	0.999	0.853	0.453
						O_2^-:	2.06×10^{-3}	0.572	0.964	1.001	0.994	0.531	0.531
Edinburgh	7.1	27.6	1.39	-	37	ROS:	27.5	0.879	0.756	0.956	0.990	0.619	0.574
						OH:	5.77×10^{-10}	0.949	0.979	0.542	0.999	0.937	0.481
						O_2^-:	4.21×10^{-3}	0.867	0.731	1.001	0.990	0.580	0.581
Toronto	12.7	55	2.5	-	111	ROS:	44.0	0.880	0.789	0.953	0.990	0.647	0.599
						OH:	1.10×10^{-9}	0.919	0.972	0.568	0.999	0.903	0.473
						O_2^-:	6.71×10^{-3}	0.862	0.766	1.001	0.989	0.605	0.606
Tokyo	16.2	-	-	2.3	153	ROS:	92.4	0.912	0.816	0.989	0.995	0.690	0.677
						OH:	1.69×10^{-9}	0.646	0.903	0.827	0.997	0.583	0.412
						O_2^-:	1.57×10^{-2}	0.879	0.816	1.001	0.996	0.663	0.664
Budapest	20	430	18.4	-	116	ROS:	124.1	0.971	0.841	0.983	0.997	0.763	0.741
						OH:	4.31×10^{-9}	0.668	0.923	0.820	0.999	0.615	0.438
						O_2^-:	2.23×10^{-2}	0.890	0.846	1.001	0.997	0.699	0.700
Hong Kong	29	140	5.7	-	122	ROS:	78.6	0.901	0.831	0.947	0.990	0.702	0.644
						OH:	2.81×10^{-9}	0.872	0.967	0.608	0.998	0.851	0.464
						O_2^-:	1.20×10^{-2}	0.863	0.811	1.002	0.988	0.645	0.647
Milan	58.6	309	18	-	120	ROS:	132.5	0.983	0.830	0.947	0.990	0.774	0.711
						OH:	6.49×10^{-9}	0.833	0.959	0.653	0.998	0.806	0.464
						O_2^-:	2.10×10^{-2}	0.910	0.820	1.003	0.989	0.697	0.699
Guangzhou	104.58	66	60	-	138	ROS:	234.5	1.009	0.753	0.928	0.990	0.755	0.680
						OH:	8.42×10^{-9}	0.961	0.986	0.537	1.000	0.952	0.491
						O_2^-:	3.08×10^{-2}	0.995	0.745	1.003	0.989	0.734	0.736
Pune (India)	113.8	2090	339	-	140	ROS:	238.6	1.380	0.786	0.978	0.997	1.050	1.012
						OH:	2.81×10^{-8}	0.738	0.906	0.838	0.999	0.675	0.515
						O_2^-:	5.23×10^{-2}	1.005	0.870	1.001	0.997	0.864	0.865
Beijing	182.2	1180	70	-	142	ROS:	189.3	1.211	0.848	0.935	0.987	0.988	0.892
						OH:	2.35×10^{-8}	0.820	0.959	0.693	0.997	0.791	0.489
						O_2^-:	3.68×10^{-2}	0.953	0.867	1.003	0.989	0.783	0.787
New Delhi	276.9	1150	70	-	132	ROS:	206.9	1.230	0.856	0.906	0.982	1.020	0.882
						OH:	3.09×10^{-8}	0.859	0.970	0.645	0.997	0.839	0.490

						O_2^{-}:	3.71×10^{-2}	0.955	0.867	1.004	0.982	0.787	0.793
Peat fires (Indonesia)	640	4810	100	-	143	ROS:	148.6	1.350	0.934	0.921	0.983	1.208	1.046
						OH:	8.04×10^{-8}	0.850	0.983	0.676	0.996	0.834	0.512
						O_2^{-}:	4.36×10^{-2}	0.978	0.944	1.001	0.983	0.887	0.892

*Initial ROS concentrations were calculated assuming average PM2.5 percentages of 30 % SOA, 1.3 % iron, 0.06 % copper and 0.005% quinones unless concentrations were known.
